# Evolution and genetic architecture of sex-limited polymorphism in cuckoos

**DOI:** 10.1126/sciadv.adl5255

**Published:** 2024-04-24

**Authors:** Justin Merondun, Cristiana I. Marques, Pedro Andrade, Swetlana Meshcheryagina, Ismael Galván, Sandra Afonso, Joel M. Alves, Pedro M. Araújo, Gennadiy Bachurin, Jennifer Balacco, Miklós Bán, Olivier Fedrigo, Giulio Formenti, Frode Fossøy, Attila Fülöp, Mikhail Golovatin, Sofia Granja, Chris Hewson, Marcel Honza, Kerstin Howe, Greger Larson, Attila Marton, Csaba Moskát, Jacquelyn Mountcastle, Petr Procházka, Yaroslav Red’kin, Ying Sims, Michal Šulc, Alan Tracey, Jonathan M. D. Wood, Erich D. Jarvis, Mark E. Hauber, Miguel Carneiro, Jochen B. W. Wolf

**Affiliations:** ^1^ Division of Evolutionary Biology, LMU Munich, Planegg-Martinsried, Germany.; ^2^ Department of Ornithology, Max Planck Institute for Biological Intelligence, Seewiesen, Germany.; ^3^ CIBIO, Centro de Investigação em Biodiversidade e Recursos Genéticos, InBIO Laboratório Associado, Universidade do Porto, Vairão, Portugal.; ^4^ Departamento de Biologia, Faculdade de Ciências da Universidade do Porto, Porto, Portugal.; ^5^ BIOPOLIS Program in Genomics, Biodiversity and Land Planning, CIBIO, Vairão, Portugal.; ^6^ Institute of Plant and Animal Ecology, Ural Branch, Russian Academy of Sciences, Yekaterinburg, Russia.; ^7^ Departamento de Ecología Evolutiva, Museo Nacional de Ciencias Naturales, CSIC, Madrid, Spain.; ^8^ Department of Genetics, University of Cambridge, Cambridge, CB2 3EH, UK.; ^9^ Palaeogenomics and Bio-Archaeology Research Network, School of Archaeology, University of Oxford, Oxford, OX1 3QY, UK.; ^10^ Department of Life Sciences, MARE–Marine and Environmental Sciences Centre/ARNET–Aquatic Research Network, University of Coimbra, Coimbra, Portugal.; ^11^ Scientific and Practical Center of Biodiversity, Irbit, Russia.; ^12^ The Vertebrate Genome Lab, Rockefeller University, New York, NY 10065, USA.; ^13^ HUN-REN-UD Behavioral Ecology Research Group, Department of Evolutionary Zoology and Human Biology, University of Debrecen, Debrecen, Hungary.; ^14^ Centre for Biodiversity Genetics, Norwegian Institute for Nature Research, Trondheim, Norway.; ^15^ Evolutionary Ecology Group, Hungarian Department of Biology and Ecology, Babeş-Bolyai University, Cluj-Napoca, Romania.; ^16^ STAR-UBB Institute of Advanced Studies in Science and Technology, Babeş-Bolyai University, Cluj-Napoca, Romania.; ^17^ British Trust for Ornithology, Thetford, UK.; ^18^ Institute of Vertebrate Biology, Czech Academy of Sciences, Brno, Czech Republic.; ^19^ Wellcome Sanger Institute, Wellcome Genome Campus, Hinxton, UK.; ^20^ Evolutionary Ecology Group, Faculty of Biology and Geology, Babeș-Bolyai University, Cluj-Napoca, Romania.; ^21^ Department of Evolutionary Zoology and Human Biology, University of Debrecen, Debrecen, Hungary.; ^22^ Hungarian Natural History Museum, Budapest, Hungary.; ^23^ Zoological Museum, Moscow State University, Moscow, Russia.; ^24^ Advanced Science Research Center and Program in Psychology, Graduate Center of the City University of New York, New York, NY 10031, USA.

## Abstract

Sex-limited polymorphism has evolved in many species including our own. Yet, we lack a detailed understanding of the underlying genetic variation and evolutionary processes at work. The brood parasitic common cuckoo (*Cuculus canorus*) is a prime example of female-limited color polymorphism, where adult males are monochromatic gray and females exhibit either gray or rufous plumage. This polymorphism has been hypothesized to be governed by negative frequency-dependent selection whereby the rarer female morph is protected against harassment by males or from mobbing by parasitized host species. Here, we show that female plumage dichromatism maps to the female-restricted genome. We further demonstrate that, consistent with balancing selection, ancestry of the rufous phenotype is shared with the likewise female dichromatic sister species, the oriental cuckoo (*Cuculus optatus*). This study shows that sex-specific polymorphism in trait variation can be resolved by genetic variation residing on a sex-limited chromosome and be maintained across species boundaries.

## Teaser

Ancient haplotypes maintained via maternal inheritance govern female-limited color polymorphism in two cuckoo sister taxa.

## INTRODUCTION

The study of sexual dimorphism has a long-standing tradition in the evolutionary sciences (*1*, *2*). Less attention has been devoted to intrasexual differences in the variability of traits (sex-limited polymorphism) despite their frequent occurrence (*3*) and substantial eco-evolutionary implications (*4*, *5*). Prominent examples include aposematic defense traits against predators in *Papilio* butterflies (*6*), the reduction of sexual conflict in damselflies (*7*), adaptive mechanisms related to ecology, social conflict, or migration in hummingbirds (*8*), and androgenetic alopecia in humans (*9*).

*Cuculus* cuckoos are obligate brood parasites and a textbook example of female-limited polymorphism in which adult males exhibit monochromatic gray plumage and females are either gray or rufous in plumage (*10*). This distinctive female-limited dichromatism is observed both in the common cuckoo (*Cuculus canorus*; hereafter *canorus*), as well as in its sister species, the oriental cuckoo (*Cuculus optatus*; hereafter *optatus*), and several other species within *Cuculidae* (*11*). Similar polymorphisms transcending species boundaries are found in other taxa and often invoke forms of balancing selection including negative frequency dependency (*12*, *13*). In cuckoos, it has been suggested that dichromatic female plumage may increase fitness of the rarer morph by escaping the costly male harassment directed at the more common morph (*14*, *15*) or by deceiving host recognition and mobbing (*16*, *17*), possibly interacting with Batesian mimicry of predatory hawk features (*18*, *19*).

Sex-specific selection is expected to be most efficient for genetic variation that is controlled by the respective sex. In male heterogametic species, the Y chromosome provides a direct target for male-specific selection, whereas in female heterogametic species (all birds, including cuckoos), female-specific polymorphism should be more likely encoded on the W chromosome. The few cases, however, where the genetic basis of sex-specific polymorphism has been identified are restricted to Mendelian autosomal loci shared between sexes [butterflies: (*6*); damselflies: (*20*); reptiles: (*21*)]. Here, we characterize phenotypic variation of female-limited dichromatism in the common and oriental cuckoo, investigate its genetic basis, and reconstruct its evolutionary history.

## RESULTS AND DISCUSSION

### Variation in female plumage color maps to the matrilineal genome in *C. canorus*

**Fig. 1.**
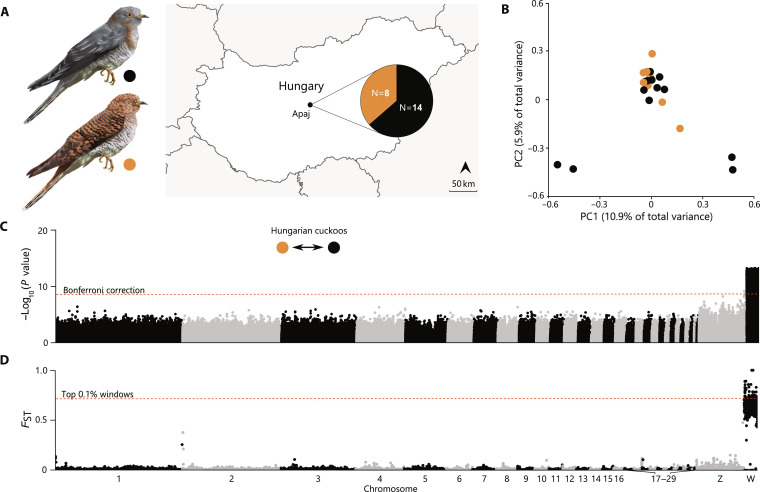
Genetic structure and mapping of female plumage polymorphism in a single population of *C. canorus*. (**A**) Representation of female gray (top) and rufous (bottom) common cuckoo plumage morphs and sampling location at Apaj village, Hungary. Map includes sample sizes from each morph. (**B**) PCA using whole-genome sequencing data showing no genetic structure between Hungarian *canorus* morphs. Individuals are colored by phenotype, and the percentage of variance explained by each component is given in parentheses. (**C**) Genome-wide association analysis. Each dot represents the −log_10_ transformation of likelihood ratio test (LRT) *P* values based on a 1 df chi-square distribution, per variant site. (**D**) Genetic differentiation between morphs across the genome. Each dot represents the fixation index (*F*_ST_) averaged in 50-kb non-overlapping windows. In (C) and (D), the significance threshold after genome-wide Bonferroni correction (*P =* 2.77 × 10^−9^) and the top 0.1% of the empirical distribution are shown by a red dashed line, respectively.

To determine the genetic basis of female cuckoo plumage coloration, we first carried out whole-genome resequencing of 22 female common cuckoos exhibiting gray (*n* = 14) or rufous plumage (*n* = 8) (table S1). Given the confounding effects of population structure for genetic mapping, we sampled these females within a single locality in Hungary (Fig. 1A, sympatric *canorus* hereafter) where all cuckoos parasitize a single host, the great reed warbler (*Acrocephalus arundinaceus*) (*15*). Individuals were unrelated (average *rxy =* 0.001) except for a single pair with a first-cousin relationship (fig. S1).

Population resequencing data were mapped against a chromosome-level assembly including the female heterogametic W chromosome generated for this study and the Vertebrate Genomes Project [National Center for Biotechnology Information (NCBI) version GCA_017976375.1_bCucCan1.pri; for summary statistics, see table S2]. We began by investigating population structure using principal components analysis (PCA). This analysis did not reveal any signature of population substructure associated with female color (Fig. 1B), as gray and rufous *canorus* appeared intermingled with no significant differences between groups (multivariate analysis of variance, *P* = 0.64). The overall lack of structure associated with dichromatism was corroborated by admixture analysis (fig. S2). These results show that the phenotypic differences between gray and rufous cuckoos were not associated with general genome-wide differentiation in a single interbreeding population and are thus amenable for genetic mapping of candidate genomic regions associated with the color polymorphism.

Next, to identify genomic regions associated with the color polymorphism, we performed genome-wide association analysis using a final filtered dataset of 5,507,428 single-nucleotide polymorphisms (SNPs). The top 99.8% of all 13,042 variants with statistical support (*P* < 2.77 × 10^−9^) were all located within the W chromosome (Fig. 1C). Shuffling phenotypic assignments indicated a robust genotype-phenotype association, indicating that the observed trends are specific to the plumage comparison, irrespective of the W chromosome’s diminished effective population size and increased potential for differentiation (fig. S3). In addition, we searched for genomic regions of elevated genetic differentiation between gray and rufous morphs using the fixation index *F*_ST_ with a sliding window approach. Genome-wide autosomal mean *F*_ST_ between the two morphs was low (*F*_ST_ ± SD = 0.01 ± 0.08), while the heterogametic female W chromosome exhibited high genetic differentiation (*F*_ST_ ± SD = 0.63 ± 0.12) (Fig. 1D). The W chromosome harbored 99.5% of the highly differentiated 50-kb windows (*F*_ST_ > 0.5; *n* = 382), with the remaining hits localized within a misassembly near the putative pseudo-autosomal region on the Z chromosome (fig. S4). Our findings thus suggest that the color polymorphism in *canorus* is genetically controlled by the maternally restricted genome [i.e., W chromosome, mitochondrial DNA (mtDNA)]. This result provides additional evidence supporting the importance of degenerated non-recombining sex chromosomes in orchestrating nonreproductive phenotypic traits (*22*), wherein the maternal components are inherited as a complete block, analogously to the autosomal control of sex-limited phenotypic traits via supergenes in other avian species (*23*).

### Plumage coloration has the same spectroscopic properties in *C. canorus* and *C. optatus*

**Fig. 2.**
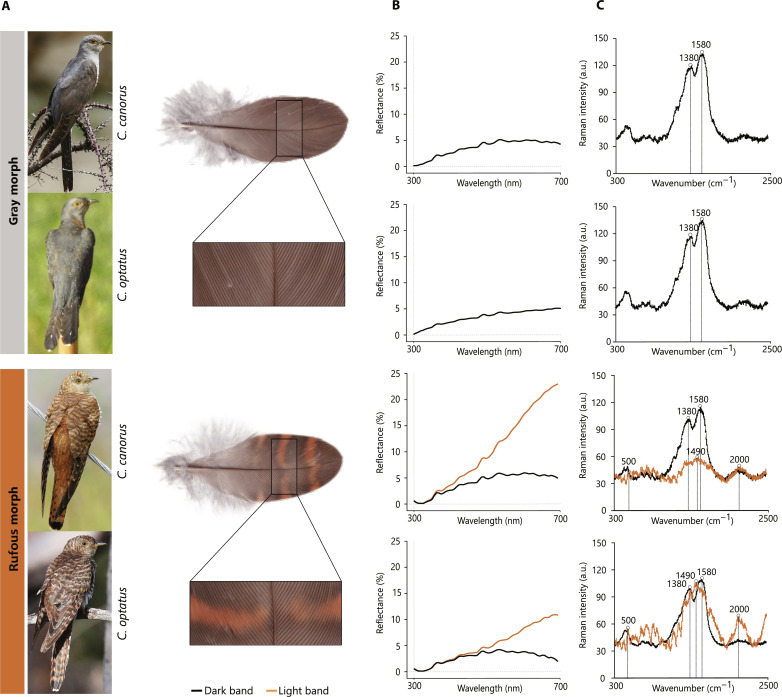
Physical and biochemical characterization of plumage color in two cuckoo species. (**A**) Photos depicting the two female plumage morphs of both species studied (common cuckoo, *C. canorus*; oriental cuckoo, *C. optatus*) (left). Feather insets show measured sites for both spectroscopic analyses (right). (**B**) Spectrophotometric measurements (percent reflectance) obtained from wing covert feathers of rufous and gray morphs for each species. (**C**) Raman spectra of the same feathers used for spectrophotometry. Diagnostic intensity peaks for eumelanin and pheomelanin identified in each spectrum of dark and light bands, respectively. (B) and (C) In rufous feathers, light and dark bands were measured separately to better account for patterning. Photo credits: Imran shah (*canorus* gray and rufous, licensed under CC BY-SA 2.0), ming110 (*optatus* gray, licensed under CC BY-NC 4.0), and Ged Tranter (*optatus* rufous, ML193641471). a.u., arbitrary units.

To examine whether the color polymorphism may have a common biochemical basis in both *canorus* and *optatus*, we quantified phenotypic variation in more detail. Adult female cuckoos of the gray morph resemble males exhibiting a largely uniform gray appearance which is interrupted by a black-and-white barred belly. Females of the rufous morph are cinnamon-brown in plumage and characterized by banded feathers alternating between dark and light areas (Fig. 2A). In both species, spectrophotometric measurements of wing covert feathers from 12 adult female birds revealed that gray morph feathers and the dark bands of rufous feathers had uniform reflectance across the whole spectrum of avian-visible wavelengths. In contrast, in the light bands of rufous feathers, the profile was skewed toward longer wavelengths consistent with their lighter and redder hues (Fig. 2B). Melanin-based color patterns in vertebrates are often produced by the differential deposition of two major pigment types: eumelanin (a brown-to-black pigment) and pheomelanin (a light-brown or reddish pigment) (*24*). To characterize differences between adult female morphs in the chemical composition of melanin-based pigmentation, we used Raman vibrational spectroscopy on the same wing covert feathers. In both species, gray feathers had a spectrum characterized by a bimodal pattern with peaks at 1380 and 1580 cm^−1^—a profile indicative of the presence of eumelanin (*25*). In rufous feathers, the dark bands resembled the spectrum of gray-morph feathers, whereas the lighter bands had a single broad peak at 1490 cm^−1^, with two satellite peaks near 500 and 2000 cm^−1^—a profile expected in regions enriched for pheomelanin (Fig. 2C) (*25*). While Raman signal profiles were near-identical across our two focal species, pheomelanin signal intensity was twofold higher in *optatus* than in *canorus* feathers, indicating higher pigment density. Overall, these results suggest that eumelanin is enriched in gray feathers and the dark bands of rufous feathers, whereas pheomelanin is enriched in the lighter bands of rufous feathers. Adult female color polymorphism may thus have a common biochemical basis across the two cuckoo species with some slight differences in color intensity likely arising from species-specific rates of pigment deposition.

### Trans-specific, female-limited color polymorphism has a common genetic basis

**Fig. 3.**
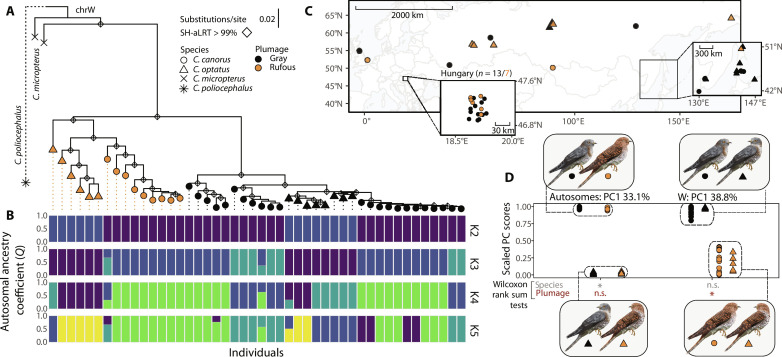
W chromosome–encoded female-limited polymorphism predates speciation of *C. canorus* and *C. optatus*. (**A**) Maximum-likelihood phylogeny of the W chromosome (*n* = 53,712 biallelic SNPs) rooted along the truncated outgroup branch of the lesser cuckoo (*C. poliocephalus*), further including two specimens of another outgroup species (*C. micropterus*). Nodes with SH-like approximate likelihood ratio test (SH-aLRT; > 99%) support are marked with a diamond. (**B**) Autosomal ancestry coefficients (*n* = 16,031,301 biallelic SNPs) with individuals (*x* axis) displayed in the same order as the phylogeny. (**C**) Jittered sampling distribution with insets for two localities with higher sampling density. (**D**) First principal component of autosomal (left) and W chromosome (right) genetic variation, with PC scores scaled from 0.0 to 1.0 (outgroups *C. poliocephalus* and *C. micropterus* excluded). Because of deviations from normality, two Wilcoxon rank sum tests were conducted on each axis to evaluate significant differences between species and plumage morphs, denoted below each axis after Bonferroni correction. The eigenvalue proportion of variance explained is indicated adjacent to each PC label. *P < 0.05; ns, not significant.

The observation that the female-limited gray-rufous dimorphism may have a shared biochemical basis between *canorus* and *optatus* (Fig. 2) raises several nonmutually exclusive hypotheses on the evolutionary origin of the phenotype (*18*). One possibility is a single source of the causal mutation originating either in the common ancestor before speciation or emerging in one of the lineages with subsequent introgression to the other species (*26*). Alternatively, similar phenotypes could be caused by independent mutations in the same or different genomic regions. To address these hypotheses, we extended the genomic analyses to *optatus* using whole-genome resequencing of samples collected from across its distributional range while supplementing additional *canorus* samples from outside of the Hungarian population (*C. optatus*: gray *n* = 8; rufous *n* = 6; *C. canorus*: gray *n* = 10; rufous *n* = 2; Fig. 3C). An *F*_ST_ outlier scan in *optatus* mirrored our observations in *canorus* (figs. S5 and S6). Genome-wide differentiation between gray and rufous *optatus* was in general negligible (mean *F*_ST_ ± SD = 0.015 ± 0.029) with the notable exception of the maternally restricted W chromosome showing strong differentiation between color morphs (mean *F*_ST_ ± SD = 0.52 ± 0.29; fig. S7). Bootstrapping analysis indicated robustness to genomic window size and sample composition excluding increased W differentiation due to population structure (fig. S8).

In agreement with strong vocalization-mediated prezygotic isolation between *canorus* and *optatus* (*27*), ADMIXTURE analyses on autosomal SNPs (*n =* 16,031,301) separated the two species into two major genetic clusters with no evidence for recent gene flow nor any association corresponding to plumage (Fig. 3B and fig. S9). The average assignment of individuals from each species to their respective cluster at *K* = 2 was never below 99.9%. For higher values of *K*, the analysis recovered substructure within each species yet never suggested any relevant genome-wide signatures of cross-species ancestry. Similarly, nonparametric statistical tests on PC1 scores separated individuals by species for autosomal data (Wilcoxon rank sum test; *P* < 1.0 × 10^−5^) but by plumage phenotype for the W chromosome (*P* < 1.0 × 10^−5^; Fig. 3D and table S3). Further extensive sampling of *canorus* museum specimens corroborated these results (total *C. canorus:* gray *n* = 29; rufous **n* =* 18; figs. S10 and S11).

**Fig. 4.**
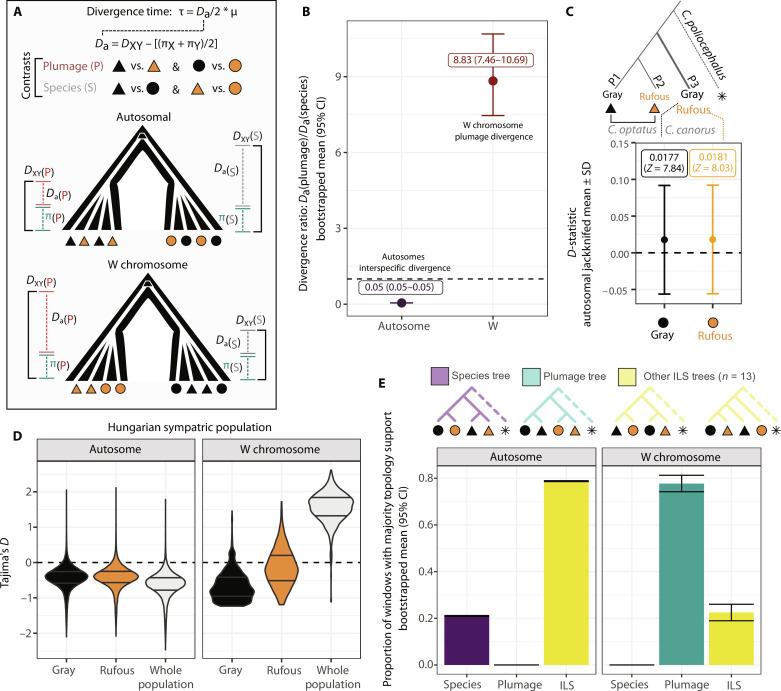
Evolutionary history of the W encoded female-limited polymorphism. (**A**) Cartoon depicting strategy for calculating age in generations since divergence using genetic variation within each population (π) and sequence divergence between them (*D*_XY_) to estimate *D*_a_ in conjunction with a generational mutation rate (μ_generation_ = 1.01 × 10^−08^). Divergence was calculated for both species split and plumage color contrast. (**B**) Relative ratio of plumage to species divergence for autosomal and W chromosomes. Mean and 95% CIs were generated by calculating the mean *D*_a(Plumage)_/*D*_a(Species)_ with 1000 bootstrap resampling events. Dashed line indicates a value of 1.0 where divergence between plumage morphs is equal to divergence between species. (**C**) Autosomal *D*-statistics from ABBA-BABA analyses. Mean and SD *D*-statistics were obtained by jackknifing all autosomal data. The analysis was run once with gray *canorus* as the target population (P3) and again with rufous *canorus* as the target (P3) to examine excess allele sharing with rufous *optatus* (P2), using *canorus* from the Hungarian population. (**D**) Tajima’s *D* in 50-kb windows for autosomal and W chromosome data within the *canorus* Hungarian dataset, with violin plots showing all windows with 25 and 75% quartiles indicated. (**E**) Proportion of genome-wide topologies exhibiting majority support (≥50%) for either the Species, Plumage, or “other ILS” tree with 100-SNP windows. All topologies other than the species tree and plumage tree were categorized as other ILS trees. Bootstrapped mean and 95% CIs are indicated.

These analyses suggest that *canorus* and *optatus* form two distinct evolutionary entities with independent autosomal histories. Yet, both appear to harbor ancient color-related, maternal haplotypes predating species divergence. Reconstructing the phylogenetic history of the female-limited W chromosome (*n =* 53,712 SNPs) supported this trans-specific polymorphism hypothesis: rufous- and gray-associated maternal haplotypes clustered by ancestry predating speciation (Fig. 3A). To shed light on the relative age of color-associated haplotypes, we quantified the time to the most common ancestor of each color morph and related it to the time of species split (Fig. 4A). Autosomal data placed speciation time between *canorus* and *optatus* at 47,679 to 52,363 generations ago [95% confidence interval (CI); Fig. S12 and table S4]. Assuming a generation time of 2.76 years (*28*), this corresponds to approximately 140 ka ago (132 to 145 ka). To obtain an age of the color polymorphism relative to speciation, we calculated the ratio of sequence divergence between color morphs and species taking differences in autosomal and W chromosomal mutation rates into account (*D*_a_ plumage/*D*_a_ species; Fig. 4, A and B). A near-zero autosomal divergence ratio (95% CI D_a_ plumage/*D*_a_ species: 0.051 to 0.053) supports the genomic entities of *canorus* and *optatus* as distinct species with negligible variation corresponding to dichromatism, while the W chromosome exhibited eightfold higher divergence between morphs than observed between species (95% CI *D*_a_ plumage/*D*_a_ species: 7.46 to 10.7). Extrapolating from autosomal speciation time places coalescence of dichromatic gray and rufous haplotypes at over 1 million years ago (0.985 to 1.55 Ma), which was further corroborated with Bayesian divergence estimation (fig. S13).

### Population genetic maintenance of the ancestrally shared polymorphism

The maintenance of trans-species polymorphism often invokes forms of balancing selection (*12*, *29*). For young species pairs, neutral processes such as incomplete lineage sorting (ILS) where ancestral gene copies by chance fail to coalesce by species provide an alternative explanation. ILS is expected to dominate patterns of ancestral variation during the early stages of species divergence (*30*) (*N*_e_ generations < 1.0; see figs. S12 and S14). Consistent with this expectation, a topology weighting analysis indicated weak genome-wide support for the species tree (mean ± SD = 33.8% ± 26.5; figs. S15 and S16 and table S5). Merely a quarter of the 173,231 autosomal genomic windows (*n* = 36,355) were assigned species topology weight ≥ 50% across all samples (Fig. 4E and table S5). However, while ILS prevailed, not a single autosomal window showed majority support for the dichromatic topology observed for the W chromosome which only had maximum observed subtree weight of 24.9% across all autosomal windows (mean ±SD = 2.2% ± 3.0; Fig. 4E and table S5). It is thus highly improbable that the observed dichromatic topology on the W, consistent across the phenotypes of all samples, occurred merely by chance. If anything, we would expect the W chromosome to be enriched for the species topology due to its reduced effective population size (haploidy and matrilineal inheritance) accelerating species coalescence.

We subsequently tested a possible contribution of adaptive introgression as an alternative explanation for trans-specificity. Near-zero *D* statistics of ABBA-BABA analyses between *canorus* and *optatus* provided little evidence for interspecific autosomal gene flow (mean ± SD *D* = 0.018 ± 0.074; Fig. 4C). We only observed slight excess allele sharing between *canorus* rufous and *optatus* rufous (*Z*_*C. canorus* rufous_ = 8.03, Z_*C. canorus* gray_ = 7.84; table S6). While these results do not exclude a contribution of gene flow between geographically adjacent ancestral populations among our rufous samples, a scenario of recent adaptive introgression of color phenotypes invoking a bottleneck and a subsequent selective sweep is highly unlikely for several reasons. Genetic diversity of W haplotypes was not depleted in either of the species (mean π *canorus/optatus*: rufous 2.0 × 10^−4^/3.0 × 10^−4^; gray 2.0 × 10^−4^/1.0 × 10^−4^; fig. S12 and table S7), Bayesian skyline plots indicate similar histories of shared expansion for rufous and gray haplotypes in both species (fig. S14), and there was otherwise no evidence for a selective sweep indicated by negative Tajima’s *D* values (Fig. 4D) or a star-shaped genealogy (cf. Fig. 3A). Instead, we observed strongly positive Tajima’s *D* on the W chromosome for both species (mean Tajima’s *D canorus*/*optatus*: 1.34/1.11; table S7), which is in stark contrast to the negative autosomal averages indicating a history of recent population expansion (mean Tajima’s *D canorus*/*optatus*: −0.696/−0.509; table S7, see also skyline plots fig. S14). While the allele frequency spectrum can be influenced by many factors, including population structure or other selective forces acting on the W, this pattern of elevated Tajima’s *D* restricted to the W chromosome is suggestive of balancing selection maintaining color variation. This prediction is consistent with field-based evidence for negative frequency-dependent selection, wherein the rarer cuckoo morph has a selective advantage either by escaping male harassment through mimicking juvenile cuckoos (*14*) or by increasing chances of successful parasitism by tricking hosts who are accustomed to the frequent morph (*17*).

Collectively, our results suggest that trans-specific, female-limited dichromatism in the two studied cuckoo species is encoded by the maternal component of the genome and has a single origin in the shared common ancestor. Maintenance by balancing selection in the form of negative frequency-dependent selection, as has been suggested on the basis of behavioral research, seems likely (*14*–*19*). These results are reminiscent of female-limited host-egg color mimicry within the *canorus* system which similarly appears to be encoded by the maternally limited genome and maintained by balancing selection (*31*). The cuckoo system thus provides a notable example of how sexual dimorphism in trait variation can be resolved by genetic variation residing on the sex-limited chromosome. Coloration has proven an effective bridge in evolutionary genetics studies by linking genotype, phenotype, and fitness (*32*). Our results therefore raise the intriguing possibility that numerous other sex-limited but less tractable traits may have similar genetic architectures to the color polymorphism of female parasitic cuckoos, wherein sex-limited chromosomes might play a substantial contributing role.

## MATERIALS AND METHODS

### Genome assembly

We generated a chromosome-level assembly of a rufous female common cuckoo (*C. canorus*) captured near Hodonín, Czech Republic (48.856°N, 17.068°E) using a combination of PacBio Sequel II CLR, 10x Genomics linked reads and Bionano Genomics Direct Label and Stain (DLS) following the Vertebrate Genomes Project (VGP) assembly pipeline v1.5 (*33*). Because of insufficient material from this individual, blood from an additional female individual sampled from the same population was used for scaffolding using Arima Genomics Hi-C (table S1). FALCON v2.0.2 and FALCON-Unzip v8.0.1 assembly algorithms were used to generate the primary contigs for the initial haploid assembly and separated the alternate haplotypic variants (*34*), followed by residual haplotype duplication processing with purge_dups v19-08-10 (*35*). Scaffolding was done in three rounds, first with 10× linked read data using scaff10X v2.0.3 (*36*), then with Bionano optical maps with Bionano Solve (Bionano Genomics, version 3.3), followed with Hi-C data generated using Arima kits (Arima Genomics) and Salsa2 HiC v.2.2.0 software (*37*). For polishing, Pacbio reads were aligned to the primary assembly with pbalign v0.3.2 and pbgcpp v1.9.0. This was followed by two additional rounds of polishing using 10× linked reads, which were aligned with Longranger Align v2.2.2.1 and variants were called with freebayes v19-07-11 (*38*, *39*). The final primary (Bioproject PRJNA562015) and alternate assemblies (Bioproject PRJNA562016) were checked for contamination, and manual curation was conducted on the primary assembly with the gEVAL v2021-03-30 (*40*) to fix structural errors, anchor scaffolds to chromosomes, and verify coherence. Further manual curation using Hi-C reads was used to guide assembly resolution, visualized with HiGlass and pretext (*41*, *42*). Manual curation resulted in 142 scaffold breaks and joins and 26 removals of erroneous duplications. This led to a 1% reduction of assembly size, 62% increase in scaffold N50, and 28% reduction in scaffold count. A total of 99.2% of the assembled sequence was assigned to 39 autosomes plus W and Z heterogametic sex chromosomes.

Final assembly contiguity was assessed with HiC-Pro v3.1.0 in 150-kb windows genome-wide (fig. S17) (*43*), and repeats were annotated with RepeatMasker 4.1.2-p1 (*44*) using a repeat library generated using Repeatmodeler v2.0.3 (*45*). Repetitive content was lower in autosomal sequence (12.4%) than in the Z (16.1%) or W chromosome (63.7%), corresponding to an excess of accumulated long terminal repeats on the W (32.4%). We annotated the assembly using transcriptome RNA sequencing (RNA-seq) data from 11 tissues collected and stored in RNAlater from a fatally injured male juvenile *C. canorus* caught in Pinhel, Portugal (40.784°N, −7.050°E), generating a total of 809 M mRNA reads (242 Gb). In short, mRNA was isolated using magnetic beads and randomly fragmented, followed by cDNA strand synthesis purification with AMPure XP beads (Beckman Coulter), end-repair, size selection, library checking with an Agilent 2100 Bioanalyzer (Agilent Technologies), and sequencing with paired-end 150 reads on an Illumina NovasSeq 6000 (Illumina). mRNA sequencing reads are deposited on the Sequence Read Archive (SRA) under BioProject PRJNA614488. Genome annotation was completed using the standard RefSeq eukaryote pipeline (BioProject PRJNA939222) (*46*).

### Maternal genomes

In this study, we tested the hypothesis of maternal encoding of adult female-limited polymorphism. In birds, females are the heterogametic sex and exclusively (co)inherit the W chromosome and the mitochondrial genome. However, nuclear insertions of mtDNA (NUMTs), which appear to be common in cuckoo (*47*), can overshadow genuine mtDNA genetic variation. We scanned the cuckoo genome for NUMTs using blastn v2.2.31+ (*48*), merging any hits found within 500 bp of one another using bedtools v2.30.0 (*49*). This search identified nine segments longer than 500 bp embedded in either autosomal chromosomes (*n =* 4) or the W chromosome (*n =* 5), including one near-complete copy on chromosome 4 (18,020 bp, 91.5% of the full-length mtDNA; table S8). Coverage parity with nuclear DNA in nucleated erythrocytes complicates separation of mitochondrial and autosomal mtDNA sources (*50*) which applies to our extensive sequencing of blood libraries. Consequently, we limited our analyses to more confident topologies and inferences from the W chromosome which includes substantially more sites. Possible confounding effects from gametologs on the Z chromosome and exclusive female W sequence were identified using coverage.

We determined reliable and unique W, Z, and mtDNA sequence for analyses involving these chromosomes by leveraging coverage discrepancies using an external dataset including male samples. Masks were computed by first calculating coverage in 100-bp windows for both chromosome W and Z for all samples and 25 bp for mtDNA using mosdepth v0.3.3 (*51*) and bedtools v2.30.0 (*49*). For the heterogametic W chromosome, we removed any windows which had male coverage (>1×) or windows where >25% of female samples exhibited coverage greater than their autosomal average (median genome wide; indicating likely repetitive sequence), masking a total of 4.10 Mb of the W chromosome. Similarly, for the Z chromosome, any regions which had more than double the autosomal average in >25% of males were masked or regions which exhibited coverage greater than the autosomal average in >25% of female samples, masking 1.84 Mb of the Z chromosome. mtDNA was masked similarly for both sexes by masking any windows exhibiting greater than double or less than one-third of the sample’s average mtDNA coverage, masking 4150 bp. Notably, these coverage masks will fail to mask any misassembled W sequence embedded within the Z chromosome. Prompted by *F*_ST_ outliers on the distal end of the Z chromosome, we subset five male samples from an external dataset and all females from the trans-species dataset (see below) to calculate median coverage in 1-kb windows using mosdepth v0.3.3 (*51*). We then normalized the coverage estimates across samples at each window scaled to the library with the fewest aligned reads, added a small constant to avoid fatal division by zero (0.01), and calculated log_2_ fold change between the mean female and mean male coverage estimates. We then visualized *F*_ST_ and log2(*F/M*) coverage using karyoploteR (fig. S4) (*52*).

### Sample collection

Our sample collection spanned three sampling regimes including live-capture and historical specimens with known plumage phenotype that were used for different analyses: 1) a sympatric dataset composed of specimens collected from a single *C. canorus* population in Hungary excluding any confounding geographic effects but including both color morphs and parasitizing the same host species, the great reed warbler *A. arundinaceus*; 2) a trans-species dataset with broad *C. canorus* and *C. optatus* sampling (including the *Hungarian* subset for most analyses) and 3) a cumulative supporting *C. canorus* dataset incorporating historical museum specimens, encompassing the widest geographic range and including additional localities with sampling from both morphs. All three sampling regimes were used for genetic analysis. Metadata for all samples is compiled in table S1 including sampling location, sex, plumage phenotype, DNA source, and permission information.

1) First, sympatric Hungarian *C. canorus* blood samples (*n* = 8 rufous and *n* = 14 gray) were obtained from live adult females near Apaj village, central Hungary (47.113°N, 19.087°E) during consecutive breeding seasons between 2014 and 2021. Birds were trapped using mist nests and conspecific playback calls as part of a long-term research project. Whole blood was drawn using standard venipuncture techniques and stored in 95% ethyl alcohol followed by salt-based DNA extraction (*53*) and library preparation with Nextera XT kits (Illumina).

2) Second, we assembled a trans-species dataset including individuals of *C. canorus* (*n* = 2 rufous and *n* = 10 gray) and *C. optatus* (*n* = 6 rufous and *n* = 8 gray) captured broadly across the Palearctic by mist netting and opportunistic collection of mortalities. The samples further included specimens from more distantly related outgroup species of *Cuculus micropterus* (*n* = 2) and *Cuculus poliocephalus* (*n* = 1). DNA was extracted from a number of different tissues (feather, muscle, and blood) using the DNeasy Blood & Tissue Kit (QIAGEN), and libraries were created with the NEBNext DNA Library Prep Kit (New England Biolabs).

3) We also generated a supporting dataset composed of 16 historical common cuckoo specimens (*n* = 10 rufous and *n* = 6 gray) from the Berlin Natural History Museum vertebrate collection (table S1). All samples consisted of dry toepad skin tissue and were extracted using an adapted version of the DNeasy Blood & Tissue Kit at a specialized ancient DNA laboratory at the University of Oxford, following a combination of standard preventive measures to avoid cross contamination. Before extraction, ~0.8 to 2 mg of skin tissue was hydrated and washed in 1× phosphate-buffered saline and EDTA solution. We used MinElute columns and added dithiothreitol (2 M) to the lysis buffer to isolate DNA fragments as short as 70 bp from degraded skin samples and optimize digestion, compensating for the low DNA yields common with commercial kits. Double-stranded whole-genome libraries were then prepared following Carøe *et al.* (*54*).

### Spectrophotometry and Raman spectroscopy

To investigate plumage color in female cuckoos, we used a combination of physical techniques. We started by describing color across the avian visible spectrum (300 to 700 nm) using reflectance spectrophotometry. For that, we collected two wing covert feathers from gray and rufous captive-bred cuckoos of two different species (*C. canorus*, *n =* 4 and *C. optatus*, *n =* 8) and stacked them against a nonreflective black surface. Reflectance measurements were taken, in replicates of five, to the left and right of the rachis for each individual. Spectra were obtained with a 400-μm ultraviolet-visible fiber optic reflection probe (Ocean Optics, USA), at a fixed distance of 3 mm and 45° angle to the feather surface. Measurements were taken using a JAZ spectrometer with an in-built PX3 pulsed xenon light source (Ocean Optics, USA) and calibrated to a diffuse 98% white standard (WS-2, Avantes BV, NL). Rufous feathers are patterned with alternating light/dark bands (Fig. 2A). For consistency, we measured reflectance of the third light/dark band (from tip), thus avoiding usually abraded and burnt feather tips. Gray feathers were measured at the same distance from tip. Spectra were processed with the pavo package (*55*) and visualized with ggplot2 (*56*), in R v4.0.3 (*57*).

Next, we characterized melanin-based pigmentation using Raman spectroscopy on the same wing coverts sample set (Fig. 2B). Raman spectroscopy is a nondestructive technique in which incident light transfers energy into molecular vibrations and the resulting spectra can be used for molecule identification (*25*). Feathers were analyzed by Raman spectroscopy to determine the relative content of eumelanin and pheomelanin, by comparing their spectra to known standards of those pigments. We used a Thermo Fisher Scientific DXR confocal dispersive microscope (Thermo Fisher Scientific, Madison, WI, USA) with a point-and-shoot Raman capability of 1-μm spatial resolution and using a near-infrared excitation laser of 780 nm. Laser power was set at 1.5 to 2 mW, integration time at 3 s, and number of accumulations at 12. The spectra were obtained using 50× and 100× confocal objectives and a slit aperture of 25 μm. The system was operated with Thermo Fisher Scientific OMNIC 8.1 software. Calibration and alignment of the spectrograph were checked using pure polystyrene. We analyzed two barbs and two barbules per individual feather, obtaining one melanin Raman spectrum from each barb and barbule. For rufous feathers, two barbs and two barbules were measured within each alternating band. Then, we computed the average Raman spectrum for each sample (species, morph, and band) and considered the three diagnostic Raman bands of pheomelanin, at 500, 1490, and 2000 cm^−1^, as described in Galván *et al.* (*58*). Similarly, eumelanin was diagnosed on the basis of 1380- and 1580-cm^−1^ Raman bands. All samples were analyzed under the same experimental conditions.

### Sequencing data generation and preprocessing

Genomic DNA quality was assessed with a combination of NanoDrop, Qubit 3.0, and agarose gels. Final libraries were assessed with either a 2200 TapeStation (Agilent Technologies), a 2100 Bioanalyzer (Agilent Technologies), or the quantitative polymerase chain reaction KAPA Library Quantification Kit (Roche). Paired-end 150-bp whole-genome sequencing was conducted on a NovaSeq 6000 (Illumina; Novogene UK) or a HiSeq X (Illumina; Macrogen, South Korea) targeting 10× coverage (minimum/mean/maximum gigabase output: 10.9/20.2/35.5 Gb). All sequencing reads are available in the Sequence Read Archive under BioProject PRJNA973617. Necessary compliance with the Nagoya protocol for sampling in each country was ensured.

Raw reads were trimmed with BBtools v38.90 (*59*) with filters for minimum read length and trimming regions falling below quality filters (-minlen 25 -trimq 2). Trimmed reads were aligned to the most recent soft-masked common cuckoo genome (see above, Refseq: GCF_017976375.1) with BWA v0.7.17-r1188 (aligned reads minimum/mean/maximum: 71.3/132/234 M) (*60*). Aligned reads were sorted with samtools v1.6 (*61*), retaining only properly paired reads and discarding reads failing platform quality checks (*-F 524*). Replicate libraries were then merged by sample and deduplicated with sambamba v0.8.1 (*62*). Reads overhanging scaffold ends were removed with GATK v4.2.4.0 (*63*), and mean coverage was calculated in 25-kb windows genome wide with mosdepth v0.3.3 (*51*). We then bifurcated our bioinformatic processing to ensure reproducibility and address the distinct objectives of each analysis. One branch used genotype likelihoods implemented in an ANGSD v0.930 framework (*64*), while the other called genotypes directly with bcftools v1.16 (*61*).

### Genome-wide association analysis

We conducted our population structure and genome-wide association analyses for the sympatric *canorus* dataset under a genotype-likelihood workflow as implemented in ANGSD v0.930 software (*64*). To compute genotype likelihoods, we restricted our calculations to biallelic positions with base quality above 30 (-skipTriallelic 1 -minQ 30) and imposed a strict threshold for a site to be considered polymorphic (-SNP_pval 1e-9*)*. We excluded reads with mapping quality below 30 and multiple best hits (-minMapQ 30 -uniqueOnly 1) and removed secondary and duplicate reads (-remove_bads 1), retaining only properly mapped reads (-only_proper_pairs 1).

To investigate population structure in our Hungarian population of cuckoos, we started by conducting a PCA using PCAngsd (Fig. 1B) (*65*). This software relies on genotype likelihood estimations from individual allele frequencies to generate a covariance matrix between all individuals. This matrix was then used to estimate principal components and individual loadings with R v4.0.3 package prcomp (*57*). In addition, we estimated individual ancestry with NGSadmix (*66*). We calculated the admixture proportion across several levels of population substructure (*K* = 2 to 5; fig. S2), imposing a minimum minor allele frequency of 0.05 (-minMaf 0.05). We also estimated the relatedness index *rxy* between all possible pairs of individuals (fig. S1) (*67*). Given population frequencies and previously computed genotype likelihoods, we inferred the relationship between individuals from their kinship coefficients using NgsRelate (*68*). PCA, admixture proportions, and relatedness index were carried out exclusively for the autosomal fraction of the genome.

To identify genomic regions associated with the different color morphs, we compared individual variants across the genomes of rufous and gray sympatric *canorus*, using a likelihood ratio test (LRT) as described in ANGSD v0.930 (Fig. 1C). In addition to the general filters described above, several stringent filters were applied to the catalog of variants before the analysis. Autosomal genotypes with less than 4× coverage and haploid genotypes (Z and W for females) with less than 2× coverage were set to missing data. Sites with >20% missing data were discarded. For SNPs detected within a 5-bp window of one another, only the SNP with the highest minor allele frequency (MAF) was retained. Last, we removed scaffolds <2 Mb in length. Association analysis was then performed on the remaining polymorphic sites.

The absence of recombination and low effective population size of chromosome W may lead to heightened differentiation of allele frequencies. We tested whether the strength of our association analysis was related to these intrinsic factors by shuffling the assignment of individuals to the control/case group with R function rand(). We did so by maintaining per-group sample size and repeatedly recalculating the LRT across the genome, over 10 independent iterations (fig. S3). Bonferroni correction was used for the significance threshold of the primary analysis (0.05/*n*, where *n =* 18,015,426; *P <* 2.77 × 10^−9^; χ^2^ = 35.34).

### Genotyping

The remainder of the analyses was based on genotype calls. SNP genotypes were called using bcftools v.1.16 (*61*), enforcing strict criteria in low mappability regions (-C 50). Similarly to the genotype-likelihood filtering, genotypes were compiled into chromosomal files and SNPs exhibiting QUAL < 20 (quality), DP exceeding twice the chromosomal average (site-level read depth), DP below the sample number, MQ < 30 (mapping quality), and read position biases (RPBZ below −3 or above 3) were excluded. Diploid chromosome genotypes with less than 4× coverage and haploid genotypes (Z and W for female samples) with less than 2× coverage were set to missing data. Weakly heterozygous genotypes on W, Z, and mtDNA were set to the major allele (binomial test of allele depth; *P* < 1 × 10^−5^) via the bcftools +setGT plugin, whereas the remaining heterozygous genotypes below this threshold were treated as missing data. Sites with over 20% missing genotypes were discarded. If multiple SNPs were detected within a 5-bp window, then only the SNP with the highest MAF was retained, implemented through the bcftools +prune plug-in. Sex chromosomes were converted to haploid genotypes, singletons were filtered where applicable (as mentioned below for each analysis), and all SNPs were then combined with invariant sites to generate an all-sites file for population genetic analyses. We analyzed bcftools stats SNP characteristics using the tidyverse in R (*57**,* *69*), calculated the mean for each statistic across samples, and examined outliers by assessing the percent difference from this mean (figs. S18 and S19). Two sympatric *canorus* samples were dropped due to low coverage (<5× genome-wide median average).

### Population genetic differentiation

We began population genetic analysis with PCAs on both autosomal and W chromosome SNPs for the trans-species dataset (excluding *C. poliocephalus* and *C. micropterus*; refer to table S1). We excluded singletons and extracted the eigenvectors and eigenvalues using plink v1.90b7 (*70*) and performed statistical analyses on the scaled (0.0 to 1.0) eigenvectors to assess differences between species (*canorus* versus *optatus*) and plumage (gray or rufous) in R v4.1.1 using the tidyverse v1.3.1 (*57**,* *69*). A preliminary inspection revealed a considerable violation of normality from a two-way analysis of variance (ANOVA), so we shifted our approach to two nonparametric Wilcoxon rank sum tests for each component (one test for each variable). We applied Bonferroni correction (*n =* 24; six components, two tests each, for both autosomes and W chromosome) and identified components associated with significant differences (*P* < 0.05) for either effect.

We next assessed autosomal structure with an ancestry analysis using ADMIXTURE v1.3.0 (*71*), using fivefold cross validation and excluding singletons as discussed above. Ancestry coefficient (*Q-*) matrices were visualized in *R* using the tidyverse and viridis packages (*57**,* *69**,* *72*). No instances of *canorus* and *optatus* with shared ancestry were observed (shared *K*) across any *K* values (Fig. 3B and fig. S9) or with expanded sampling within the supporting dataset (fig. S10). Linkage-disequilibrium SNP pruning across two species with unknown population structure deflates divergence estimates by removing diagnostic SNPs fixed between species (*73**,* *74*), so we present the full SNP panel (*n =* 16,031,301 SNPs; Fig. 3D and figs. S9 and S10). We investigated population differentiation and divergence linked to species and plumage by segregating samples into six distinct populations (sympatric Hungarian *canorus*, rufous; sympatric Hungarian *canorus*, gray; all *canorus*, rufous; all *canorus*, gray; *optatus*, rufous; *optatus*, gray; see table S1). We evaluated π and pairwise *F*_ST_ and *D*_XY_ between populations in 50-kb windows using the all-sites variant file, excluding singletons and requiring a minimum of 20% of called sites (-m 10000) using Simon Martin’s genomics general repository (*75*). Haploid chromosomes were ensured to be appropriately interpreted. We tested result sensitivity by adjusting the window size to 5 and 500 kb, maintaining the 20% called site requirement, and reached qualitatively the same conclusions (figs. S7 and S8). Genome-wide variation was visualized with karyoploteR v1.20.0 (*52*). We also examined the potential influence of sample size variation or uneven sampling on the results. This was done by randomly sampling individuals from each population (*n* = 5) and repeating the analysis across all window sizes, in 10 separate iterations. The mean, SD, and 95% CI *F*_ST_ and *D*_XY_ values were calculated in R using the tidyverse (fig. S8) (*57**,* *69*). We corroborated our π results using an additional invariant-sites aware estimator, *pixy* v1.2.7.beta1, using the same window size (fig. S20) (*76*). We confirmed that W chromosome differentiation was associated with plumage by repeating our *F*_ST_ scan 10 times using shuffled population labels (fig. S21).

We further assessed how population structure varied across the genome with a running window principal components analysis with lostruct v0.0.0.9000 (*77*) using the trans-species dataset. We statistically phased and imputed missing genotypes within variant call files (VCFs) with Beagle v5.2 and ran lostruct in 500-SNP windows using R v4.1.1*.* Running window PCA allows for the identification of shared ancestral regions without specifying discrete groups, which nonetheless identified W chromosomal windows as exhibiting unique ancestral information (fig. S22). Upon inspection, these windows exhibit population structure corresponding to plumage (fig. S22).

### Autosomal introgression

We assessed genome-wide admixture between *optatus* and *canorus* with ABBA-BABA analyses using the trans-species dataset, which detects excess allele sharing between a target group (P3) and other populations (P1 and P2) using an outgroup (O = *C. poliocephalus*). Allele frequencies from the same all-sites genotype matrix file used above for *F*_ST_ and *D*_XY_ estimation were estimated using freq.py from the genomics_general repository. Jack-knifed *D* statistics (*n* = 1070 blocks) using scripts from the same repository were estimated with *optatus* gray as P1 and *optatus* rufous as P2. We performed the analysis twice, once with *canorus* gray as P3 and once as *canorus* rufous as P3 to determine whether there was excess allele sharing between rufous *canorus* and *optatus* (excess ABBA; table S7).

ILS could provide an alternative explanation for discordant autosomal and W chromosome trees. We assessed genome-wide topologies in 100-SNP windows using Twisst (*78*), requiring at least 10 scored genotypes in all individuals to retain a window. Trees were estimated with phyml v3.3.20200621 (*79*) using a General Time Reversible (GTR) substitution model and with five population tips: *C. poliocephalus* as the outgroup, *canorus* rufous, *canorus* gray, *optatus* rufous, and *optatus* gray. Trees were summarized using scripts within the Twisst repository, wherein each 100-SNP window is assigned a topology weight ranging from 0.0 to 1.0 for the 15 possible topologies (figs. S15 and S16). Topologies were assigned as either a “Species” topology if it followed the species tree (cf. Fig. 4E), a “Plumage” topology if it exhibited the discordant plumage topology, or “ILS” for any other observed tree. Windows were assigned a majority consensus into one of the three categories if the topology weight was greater than or equal to 50%. We estimated 95% CIs using 1000 bootstrap resampling events in R using the tidyverse (*57**,* *69*).

### Phylogenetic and demographic reconstruction

Maximum-likelihood phylogenies were estimated using IQTREE v2.2.0.3 (*80*) from fasta files created from the haploid W chromosome VCF including singletons (trans-species dataset; *n =* 53,678 SNPs) using vcf2phylip.py (*81*)*.* Substitution model selection was performed with modelfinderplus (*82*) with ascertainment bias correction with final consensus tree bootstrap support estimated from 1000 ultrafast bootstraps (*83*) and 1000 bootstrap replicates for a Shimodaira–Hasegawa–like (SH-like) approximate likelihood ratio test (*79*). Phylogenies were rooted in R v4.1.1 (*57*) with phytools v1.5-1 (*84*) and visualized with ggtree v3.6.2 (*85*)*.* We additionally generated phylogenies with the same parameters for the full sample supporting dataset with stricter genotype missingness filters (supporting dataset with maximum 5% genotypes missing; *n =* 5155 SNPs; fig. S11) and for mtDNA (max-missing 20%: *n =* 383; fig. S23). Additional phylogenies were generated from the haploid Z chromosome and the diploid chromosome 6 (using International Union of Pure and Applied Chemistry (IUPAC) designations for heterozygous sites) with the same parameters and a 5% missing genotype filter (fig. S24).

We estimated the demographic history of each species and plumage morph using Bayesian Skyline Plots (*86*) using full-length protein coding gene sequence from the W chromosome (including introns and exons). Only genes with at least 10 SNPs and between 10 and 75 kb were retained for analysis, resulting in 1.47 Mb of sequence for analysis. We subset this fasta into species and plumage morph nexus files using EMBOSS v6.6.0 (*87*) and created a BEAST xml file using BEAUTi v2.6.2 (*88*). For all reconstructions, we used a gamma site model with four categories using a GTR substitution model with estimated frequencies and a per-generation strict clock rate set to half the autosomal rate (μ_generation_ = 5.05 × 10^−09^; the same as used in the other divergence date estimation analyses, see below). We ran the coalescent Bayesian Skyline model with 30 M chains using BEAST v2.6.2 (*88*), evaluated convergence with the Effective Sample Size (ESS) (table S9), and reconstructed the skyline plots with Tracer v1.7 (*89*). Skyline outputs were saved in text format and plotted with ggplot2 v3.4.1 (*56*).

We assessed departures from neutrality and signals of population expansion on autosomal, Z, and W chromosomes using Tajima’s *D*. Directional selection is associated with excessive low and high-frequency variants and negative Tajima’s *D*, while balancing selection is associated with excessive intermediate frequency variants and positive Tajima’s *D* (*90*). Tajima’s *D* in 50-kb windows was estimated using the genomics_general repository for the sympatric dataset, including singletons and run for each morph separately maintaining sample size parity (*n =* 5) as well as for the entire population (*n =* 10; Fig. 4D and table S6). Sensitivity to sampling was assessed by repeating this analysis three times with different subsampled individuals (fig. S20). Tajima’s *D* was also assessed within both *canorus* and *optatus* using the full trans-species dataset (table S7). We further ensured that these results were robust to potential population assumptions by estimating Tajima’s *D* in the Hungarian population according to the empirical rufous (60%) and gray (40%) observed morph frequencies. We achieved this by repeating the analysis 10 times with randomly subsampled rufous (*n = 7*) and gray (*n = 5*) individuals without replacement from Hungary (fig. S25).

### Divergence dating

We estimated the divergence times of each pair of populations by leveraging the statistic *D*_a_ which represents the number of mutations accumulated since the split of the most recent common ancestor (*91*), using the formula $D_{a}=D_{\mathrm{XY}}-\frac{\left(\pi_{X}+\pi_{Y}\right)}{2}$ . This method assumes equivalent levels of variation in contemporary populations and their common ancestor but still offers a relative divergence rate for comparing the non-recombining W chromosome with autosomes (*92*). Lacking empirical germline mutation rates for *Cuculiformes*, we scaled our results to generations using the avian-wide mean value (1.01 × 10^−08^ mutations per site per generation), derived from an expansive study on trios (*93*). Given the maternally restricted W chromosome and the higher mutation rates of the male germ line due to increased cell divisions, theory predicts a lower mutation rate for the W chromosome or roughly half the Z mutation rate, as seen in flycatchers (*94*). Although theoretically, the Z mutation rate should surpass the autosomal rate, given its greater exposure to the male germ line, empirical evidence is limited and any effects appear slight (*95*). Therefore, we maintained equivalent Z and autosomal mutation rates but used half the rate for the W chromosome (μ_generation_ = 5.05 × 10^−09^). Notably, no sex bias has been observed in common cuckoos (*96*).

We then calculated divergence time in generations $\tau =\frac{D_{a}}{2\mu }$ for all population pairs within the trans-species dataset for each genomic window to provide a distribution of most likely autosomal divergence estimates. We first obtained a single autosomal divergence estimate by comparing all *C. canorus* against all *C. optatus*, followed by species-morph comparisons (Fig. 4A and fig. S12). We estimated divergence of all 50-kb windows to obtain a distribution of *D*_a_ and subsequent τ for autosomal chromosomes (only considering chromosomes >10 Mb in size; fig. S12). Relative divergence between morphs (*D*_a_ plumage) and between species (*D*_a_ species) was estimated with the ratio (*D*_a_ plumage/*D_a_* species), where a value of 1.0 indicates equal divergence estimates between plumage morphs and species. We estimated mean and 95% CI *D*_a_ ratios for autosomes and the W chromosome using boot v1.3-28 in R with 1000 resampling events (*57**,* *97*). We repeated this entire workflow except including singletons and arrived at the same conclusions (fig. S12).

We corroborated our dating results using BEAST v2.6.2 (*88*)*.* W chromosome consensus fasta sequences were extracted for *C. poliocephalus* and the highest coverage *C. micropterus*, as well as the five highest coverage samples from each cuckoo morph group (*canorus* rufous and gray, *optatus* rufous and gray; *n* = 20). All non-overlapping open reading frames (ORFs) were extracted from W chromosome genes using the same fasta file as the Bayesian Skyline Plots, provided that the ORFs were shared among all 22 samples and had intact start (methionine) and stop codons (*n* = 513 ORFs). A random representative sample was chosen from each cuckoo morph group and extracted, in addition to the outgroups (*n* = 6 total individuals), to a nexus file for BEAST divergence date estimation using a Calibrated Yule model with four gamma categories and an HKY substitution model using half the autosomal mutation rate as a clock rate (μ_generation_ = 5.05 × 10^−09^). A log normal prior for the divergence between *C. micropterus* and *C. poliocephalus* was set (*M* = 1.4, *S* = 0.15) giving a 95% interval spanning 2.84 to 5.25 million years, based on estimated divergence from mtDNA data (*31*). The most supported tree was extracted and annotated with mean heights using Treeannotator with a 10% burn-in with trees and 95% highest posterior density (HPD) interval height estimates visualized with ggtree (*85*). This entire process was repeated four times to sample different representative individuals from each cuckoo morph group (fig. S13 and table S10).

### Ethical approval

All applicable international, national, and/or institutional guidelines for the care and use of animals were followed. Permission for blood sampling of wild common cuckoos was granted in the Czech Republic by the Hodonín Municipality Office (MUHOCJ 14306/2016/OŽP) with experimental design approved by the Czech Ministry of the Environment (3030/ENV/17–169/630/17) and in Hungary by the Middle-Danube-Valley Inspectorate for Environmental Protection, Nature Conservation and Water Management, Budapest (permit nos. PE/KTF/17190-3/2015 and PE/KTFO/3097-10/2020). In the UK, loose feathers from common cuckoos (*C. canorus*) were collected as a by-product of tagging work carried out under license from the Special Marks Technical Panel. Permission for sampling of blood from wild common and oriental cuckoos in Russia in compliance with the Federal Law of Russian Federation No. 498-Ф3, as ensured by the Research Bioethics Committee of the Institute of Plant and Animal Ecology, Russian Academy of Sciences. Necessary compliance with the Nagoya protocol was ensured for each country where genetic resources were obtained from.
