# Supplementary material for: Evolution and genetic architecture of sex-limited polymorphism in cuckoos
Source: Sci Adv. 2024 Apr 24;10(17):eadl5255. doi: 10.1126/sciadv.adl5255 (PMC11042743; doi:10.1126/sciadv.adl5255)
Supplement: Supplementary file 1 — Figs. S1 to S25 Tables S2 to S10 Legend for table S1 [file sciadv.adl5255_sm.pdf]

Science Advances
AAAS

Supplementary Materials for

Evolution and genetic architecture of sex-limited polymorphism in cuckoos

Justin Merondun *et al.*

Corresponding author: Jochen B. W. Wolf, j.wolf@bio.lmu.de; Miguel Carneiro, miguel.carneiro@cibio.up.pt

Sci. Adv. **10**, eadl5255 (2024)
DOI: 10.1126/sciadv.adl5255

The PDF file includes:

Figs. S1 to S25
Tables S2 to S10
Legend for table S1

Other Supplementary Material for this manuscript includes the following:

Table S1

Supplementary Figures

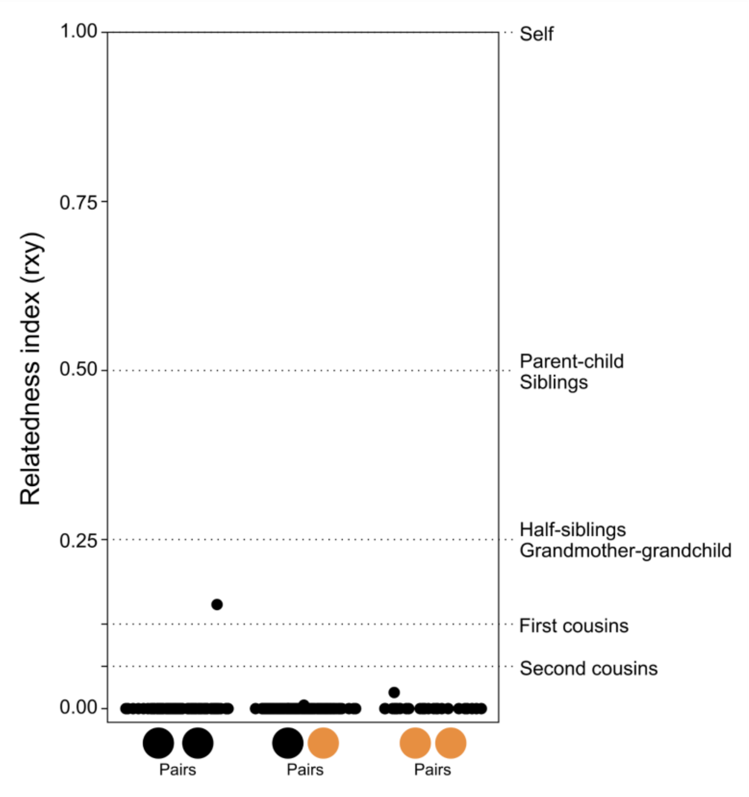

Fig. S1. Estimated pairwise relatedness among Hungarian *canorus*.

Kinship coefficients between all possible pairs of individuals (*n* = 22) within a single interbreeding population (Apaj, Hungary) were estimated from autosomal SNPs using NgsRelate. Population frequencies and genotype likelihoods computed with ANGSD v0.930.

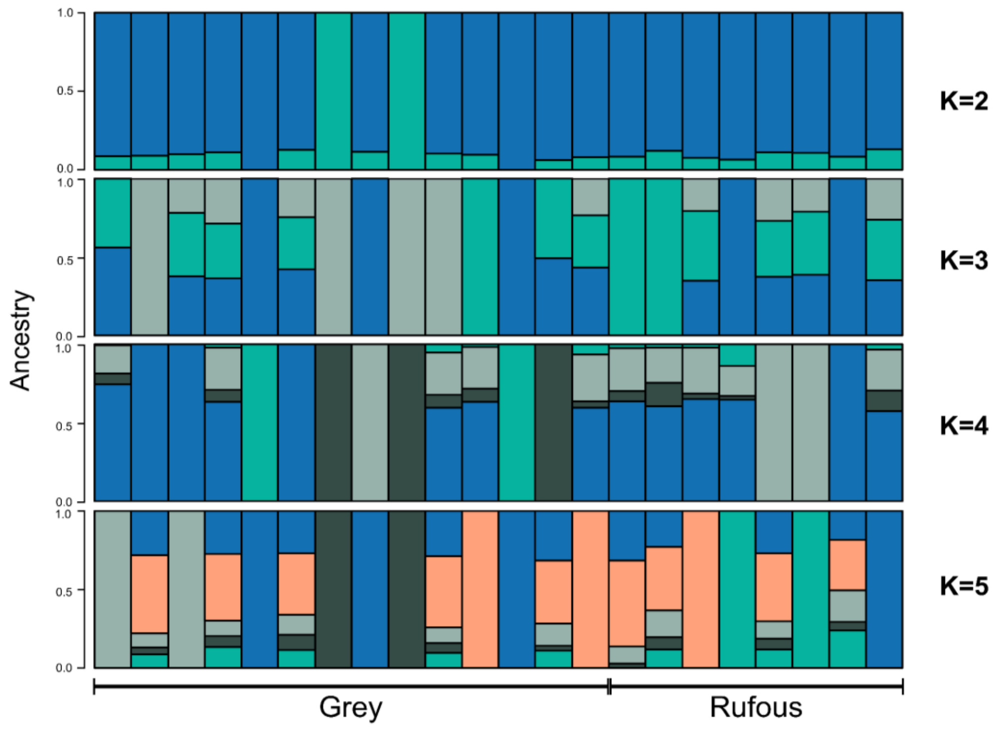

Fig. S2. Individual admixture proportions among Hungarian *canorus*.

Individual autosomal ancestry coefficients inferred from genotype likelihoods using NGSadmix. Estimates were iterated to a maximum of 2,000 times and the number of ancestral populations was set to K2 - K5. Each bar represents one individual.

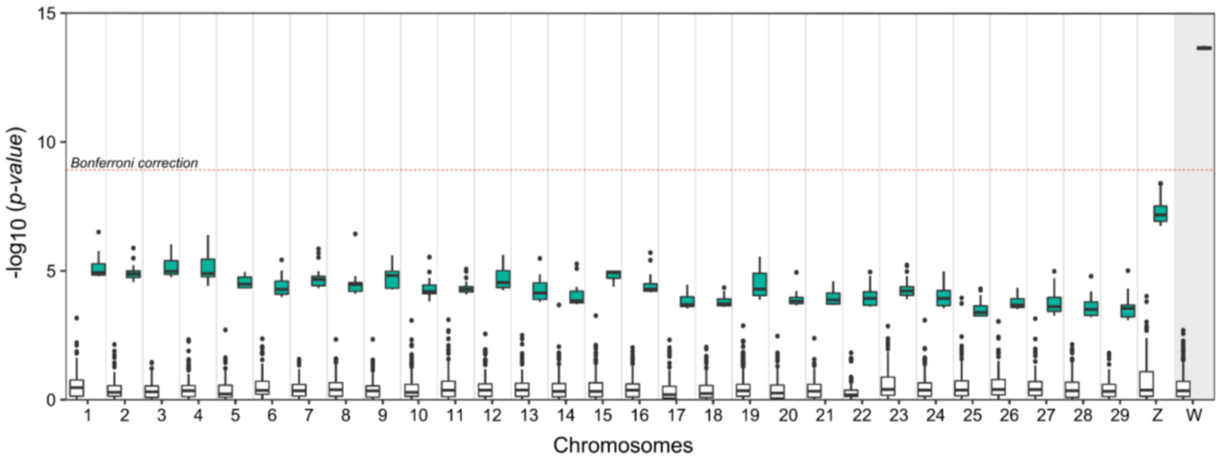

Fig. S3. Bootstrapped genotype-phenotype association within Hungarian *canorus*. Genome-wide association analysis calculated under two sample assignment schemes: in **blue**, samples assigned to control/case groups based on plumage phenotype; in **white**, samples randomly assigned to two groups, with fixed sample size, over ten independent iterations. Variant sites across the genome were compared using a Likelihood Ratio Test following a 1-*df* chi-squared distribution. Resulting *p-values* were $-\log_{10}$ transformed for visual representation. The top 20 most significant SNPs (n = 20 in **blue**, n = 20 x 10 iterations in **white**) were selected from each run and box plotted using ggplot2. Bonferroni significance threshold was set to $P > 2.77 \times 10^{-9}$.

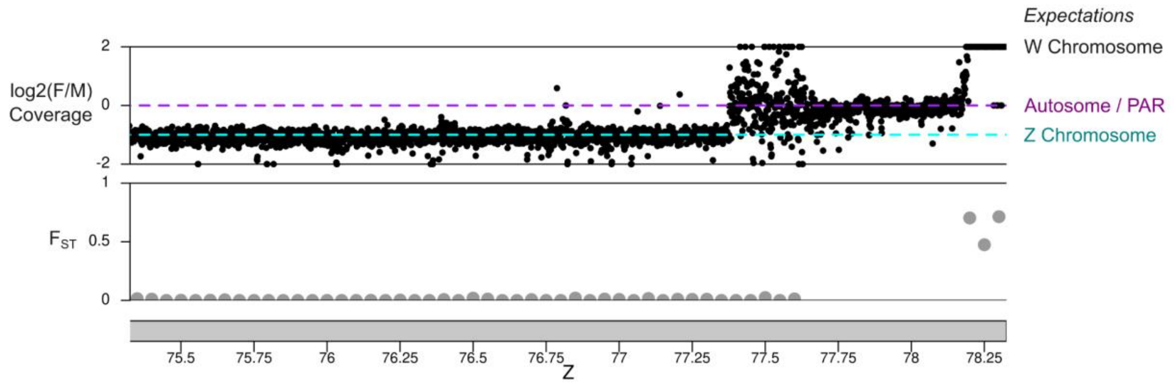

Fig. S4. Z chromosome putative misassembly.

Sex chromosomes are difficult to contiguously assemble, particularly for the repeat-laden W chromosome. Several F_{ST} outlier windows and SNPs significantly associated with plumage (LRT analyses) on the distal end of the Z chromosome prompted us to inspect female to male coverage ratios (*log2*) within this vicinity using an external dataset of five male samples in 1-kb windows using mosdepth, with all samples normalized according to the library with the minimum aligned reads. While a portion of this chromosome arm exhibits coverage levels (*log2(F/M)*) consistent with a pseudoautosomal region (PAR), several areas also exhibit W chromosome expectations (strongly female biased, approaching infinity), indicating a likely misassembly and true W sequence embedded within the Z chromosome.

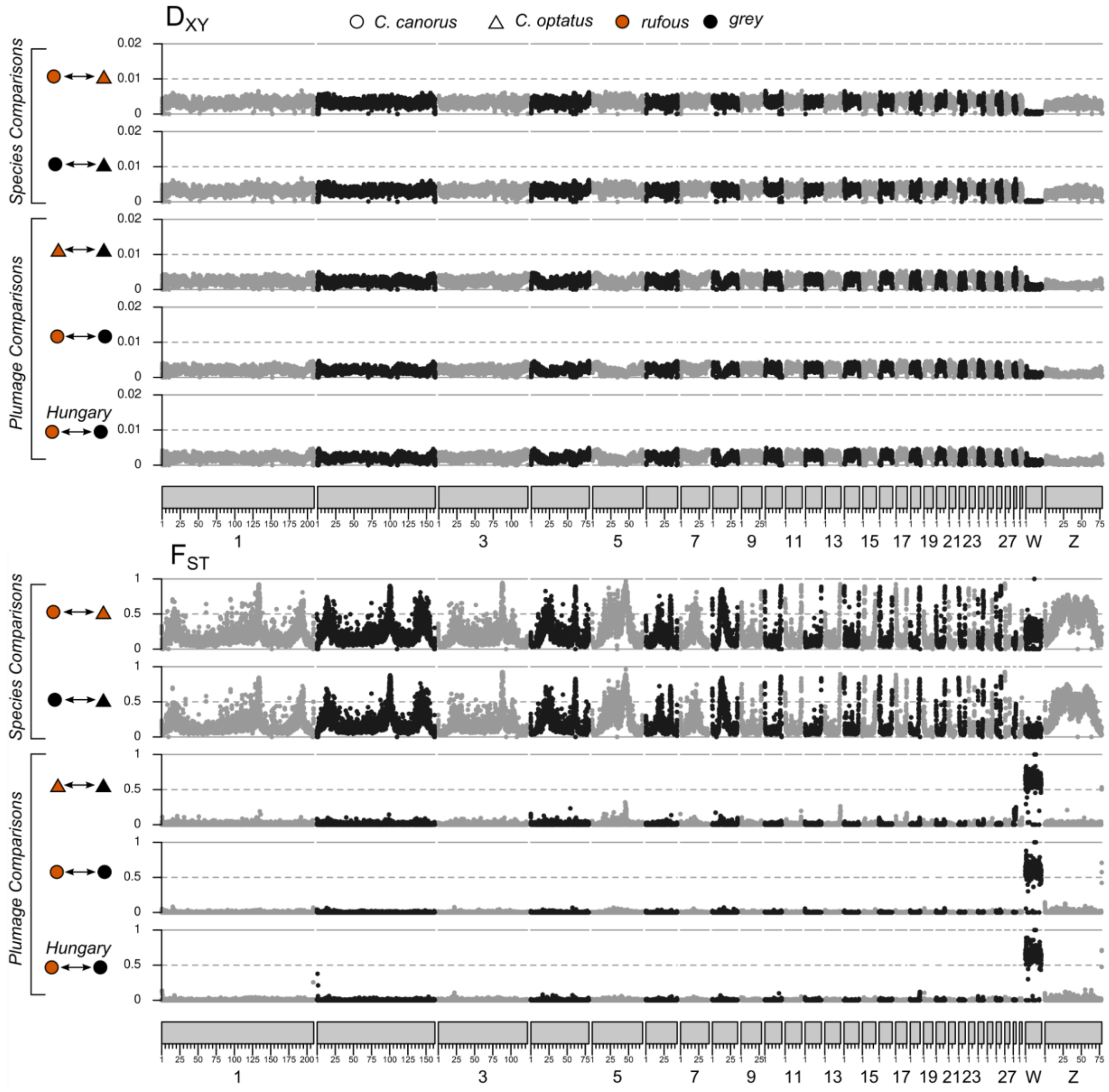

Fig. S5. Genome-wide genetic differentiation and divergence.

Pairwise genetic divergence (D_{XY}, top) and differentiation (F_{ST}, bottom) between two interspecific comparisons and three intraspecific plumage comparisons, utilizing the *Trans-species* dataset with inclusion of invariant sites. All available samples were included for analysis. Population genetic metrics were calculated in 50-kb windows, retaining only windows with a minimum of 20% valid sites. Sex chromosomes were considered haploid for genotyping and subsequent analysis.

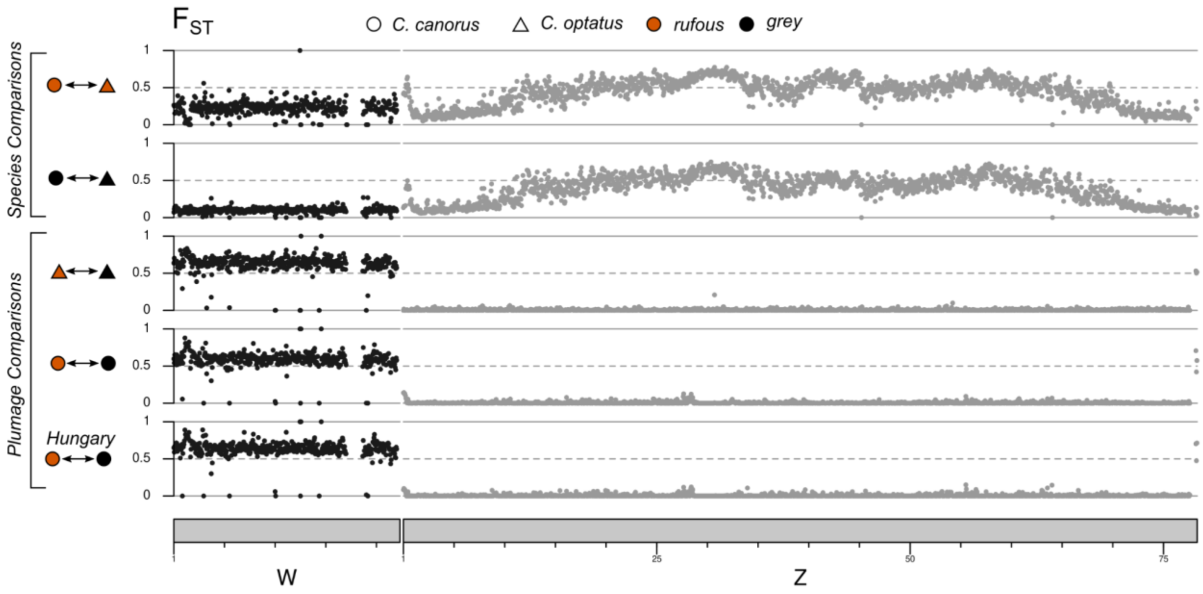

Fig. S6. Sex chromosome genetic differentiation.

Pairwise genetic differentiation (F_{ST}) between two interspecific comparisons and three intraspecific plumage comparisons, utilising the *Trans-species* dataset with inclusion of invariant sites. All available samples were included for analysis. Population genetic metrics were calculated in 50-kb windows, retaining only windows with a minimum of 20% valid sites. Sex chromosomes were considered haploid for genotyping and subsequent analysis.

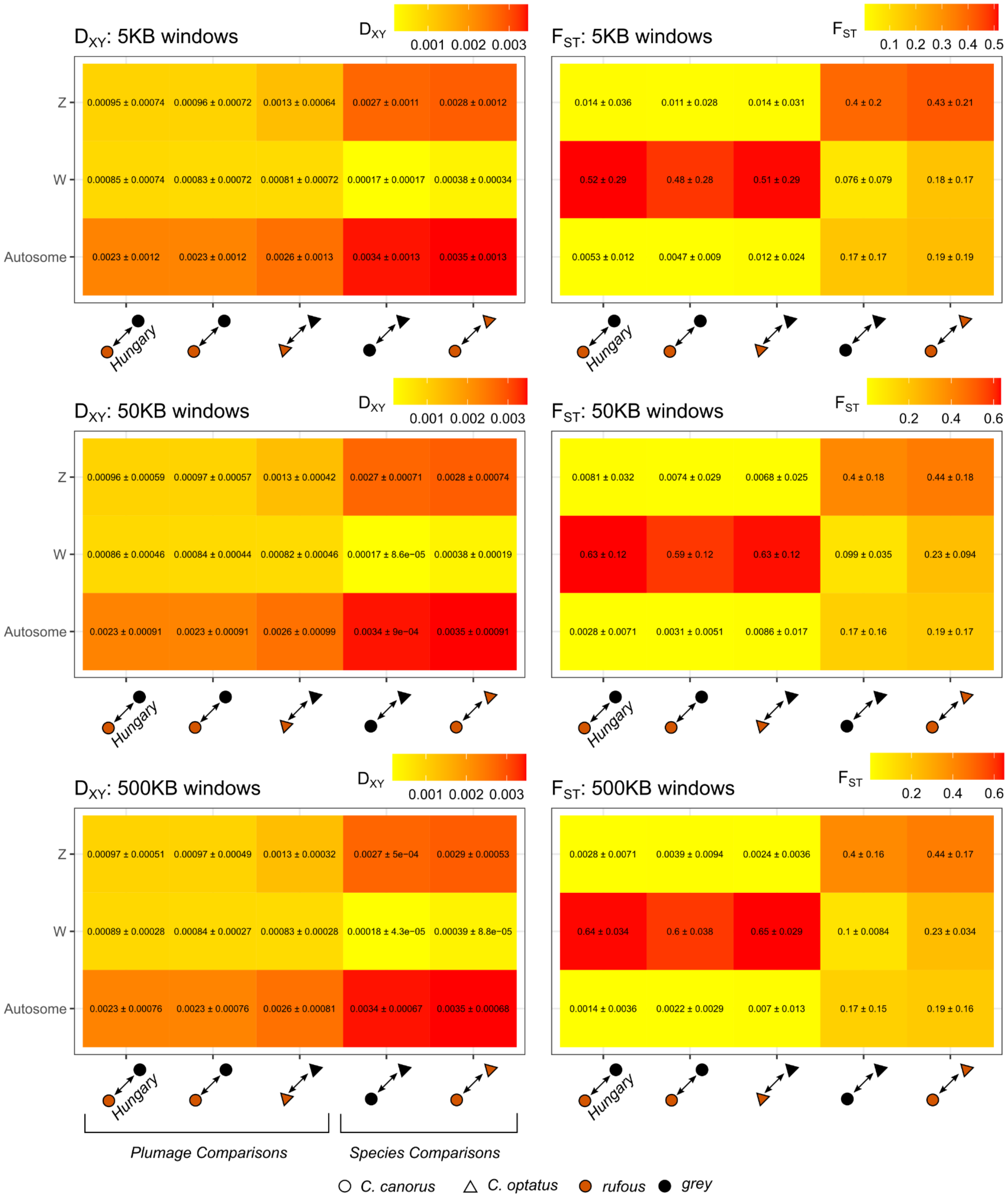

Fig. S7. Autosomal, Z, and W estimates of differentiation and divergence.

Pairwise genetic differentiation (F_{ST}, right) and divergence (D_{XY}, left) between three intraspecific plumage comparisons and two interspecific comparisons, utilising the *Trans-species* dataset with inclusion of invariant sites (mean and standard deviation indicated). All available samples were included for analysis. Vertical panels illustrate variations according to window size used in calculations, retaining only windows with a minimum of 20% valid sites. Sex chromosomes were considered haploid for genotyping and subsequent analysis.

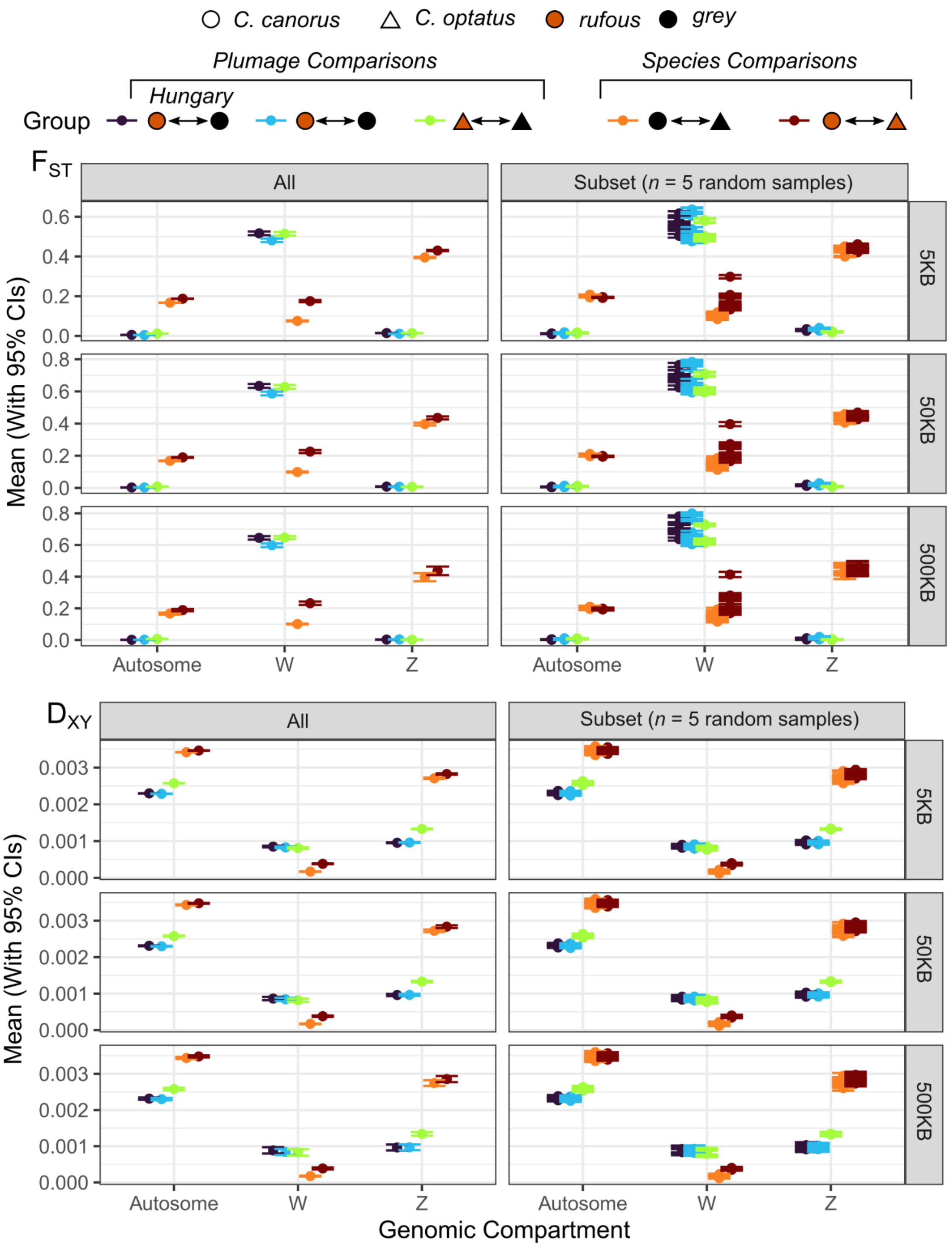

Fig. S8. Insensitivity of window size or sample selection on differentiation and divergence. Pairwise genetic differentiation (F_{ST}) and divergence (D_{XY}) between three intraspecific plumage comparisons and two interspecific comparisons (denoted by distinct colors; mean and 95% confidence intervals), utilizing the *Trans-species* dataset with inclusion of invariant sites. Left panels display comparisons incorporating all samples, whereas right panels depict ten bootstrapped iterations of comparisons using a random subsample of five samples per group. Vertical panels illustrate variations according to window size used in calculations, retaining only windows with a minimum of 20% valid sites. Sex chromosomes were considered haploid for genotyping and subsequent analysis.

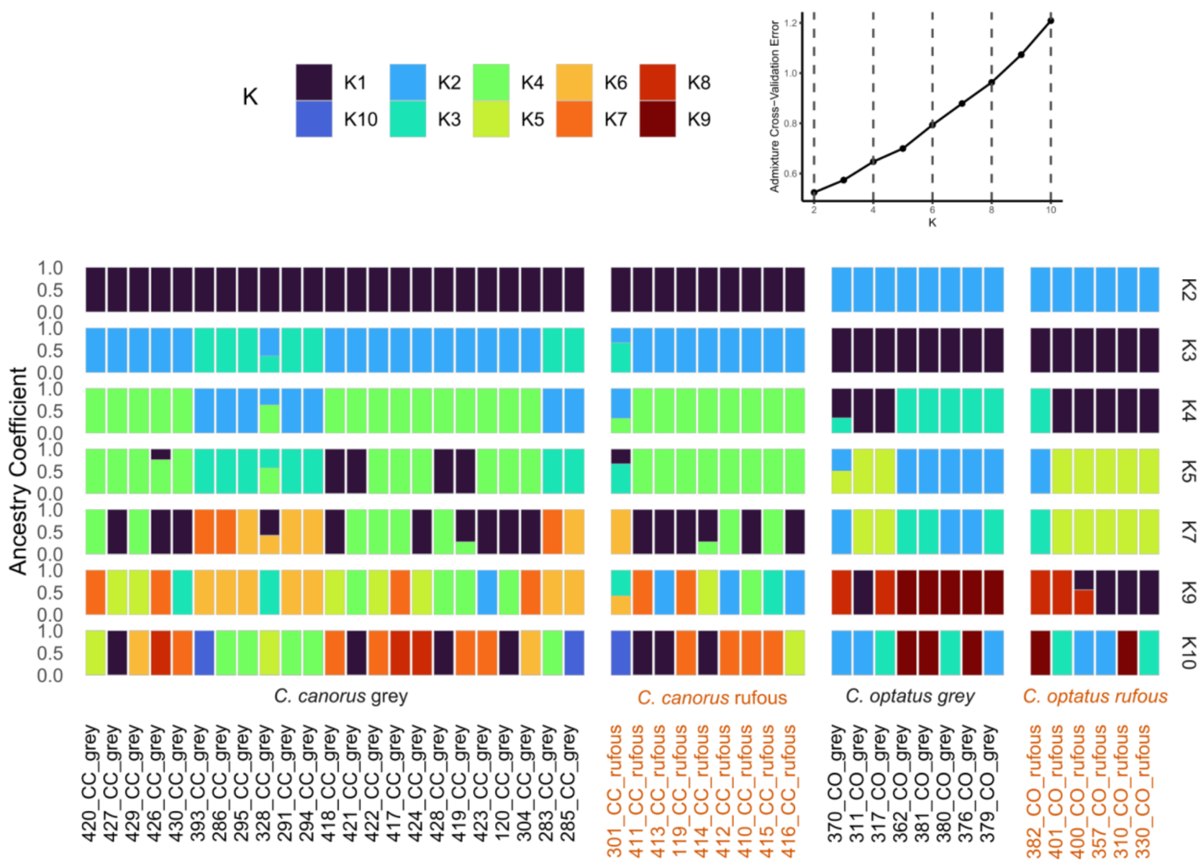

Fig. S9. Autosomal structure among *C. canorus* and *C. optatus*.

Ancestry coefficients for autosomal SNPs (*n* = 16,031,301) using ADMIXTURE v1.3.0 for additional levels between K2 - K10 for the genotype calling dataset (*Trans-species* dataset*; cf* **Fig. 3B** but showing additional *K*). Cross-validation errors (5 folds) are shown above the plot, indicating increased error with increasing *K* and supporting the genetic distinctiveness of *C. canorus* and *C. optatus* with no hybrid observations.

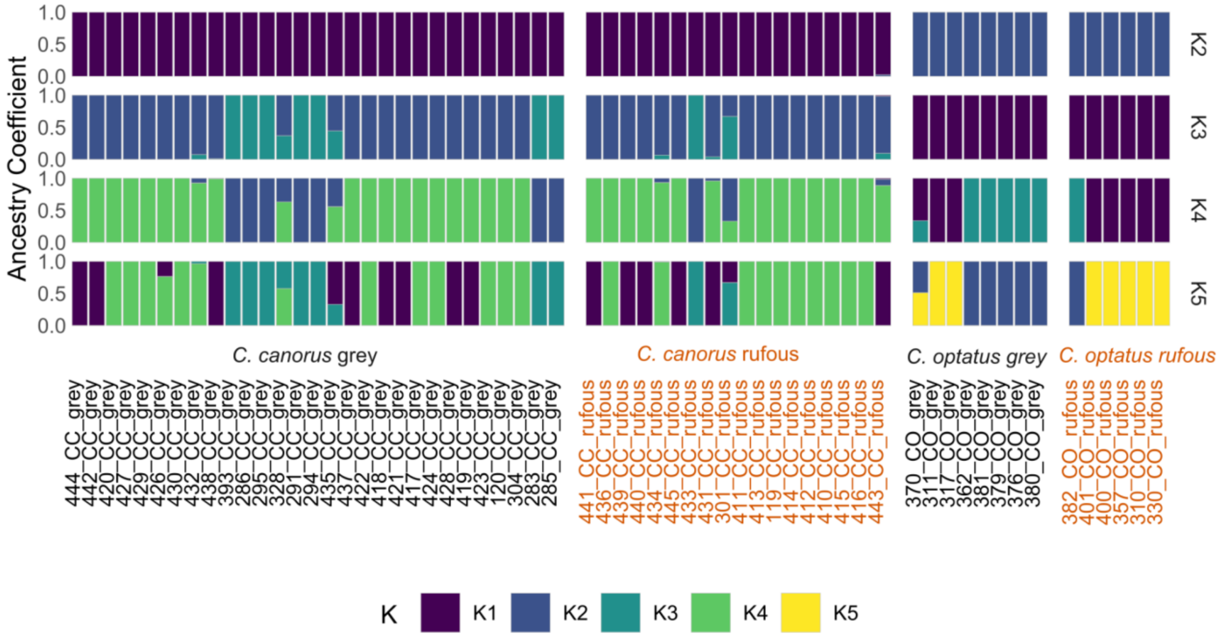

Fig. S10. Full dataset autosomal structure.

Ancestry coefficients for autosomal SNPs (n = 16,031,301) using ADMIXTURE v1.3.0 for K2 - K5 for the full genotype calling dataset (*Supporting* dataset).

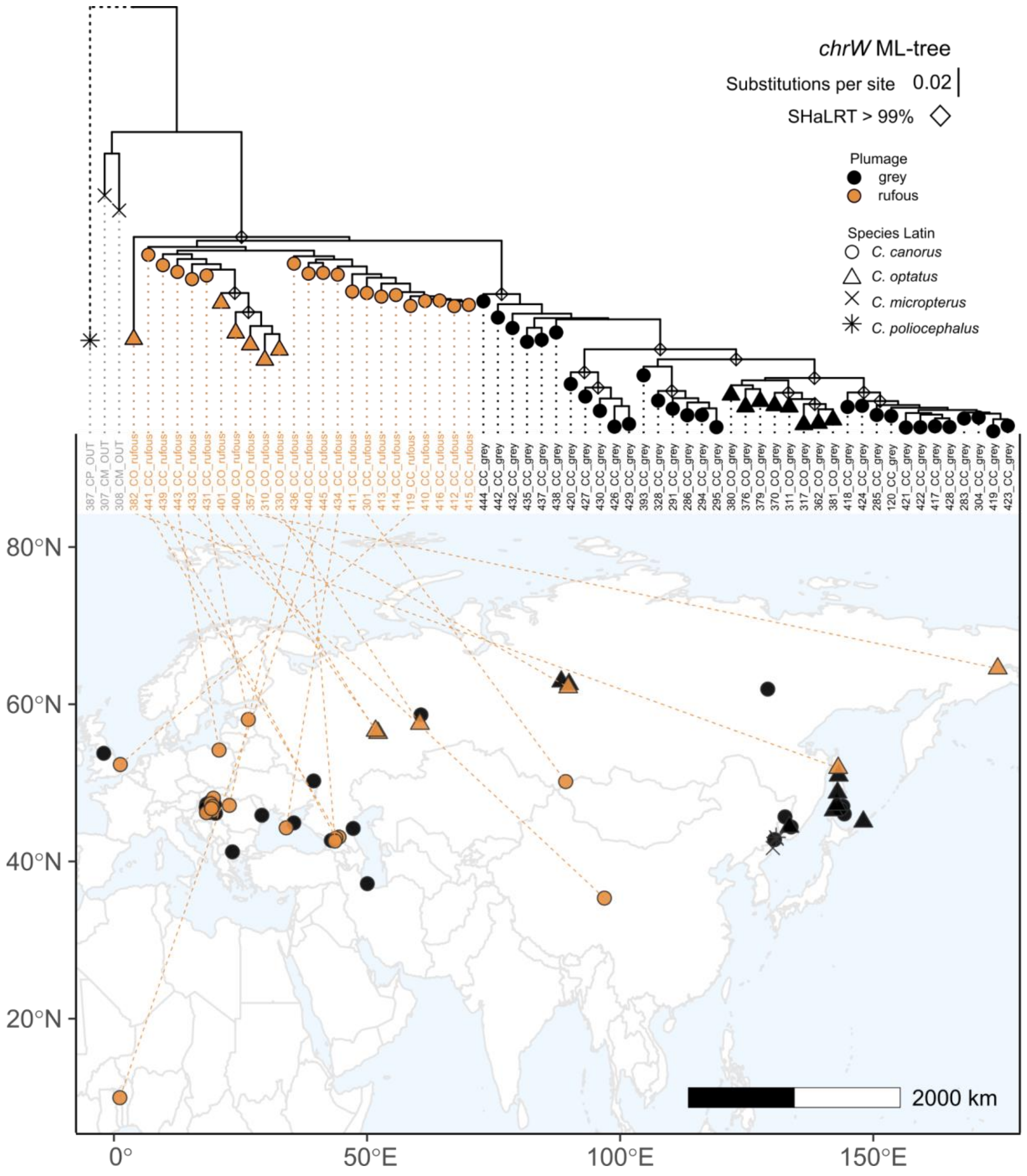

Fig. S11. Full dataset W phylogeny and sampling locations.

Maximum-likelihood phylogeny (top) from W chromosome biallelic SNPs (*n* = 5,155) using IQTREE2 with modelfinderplus and an ascertainment bias correction on the full dataset including toepad samples (*n = 64; Supporting* dataset). SNPs were removed if they failed standard INFO filters or were missing more than 5% scored genotypes, and retained only a single SNP if co-occurring within 5-bp. SNPs were also removed if they occurred within a region (25-bp resolution) violating male to female coverage expectations for a haploid chromosome (see methods). Sampling distribution (bottom) with positions jittered up to 1.0 degrees, with dotted lines connecting the occurrence locations to the labels of the phylogeny for rufous individuals (excluding *Hungary*).

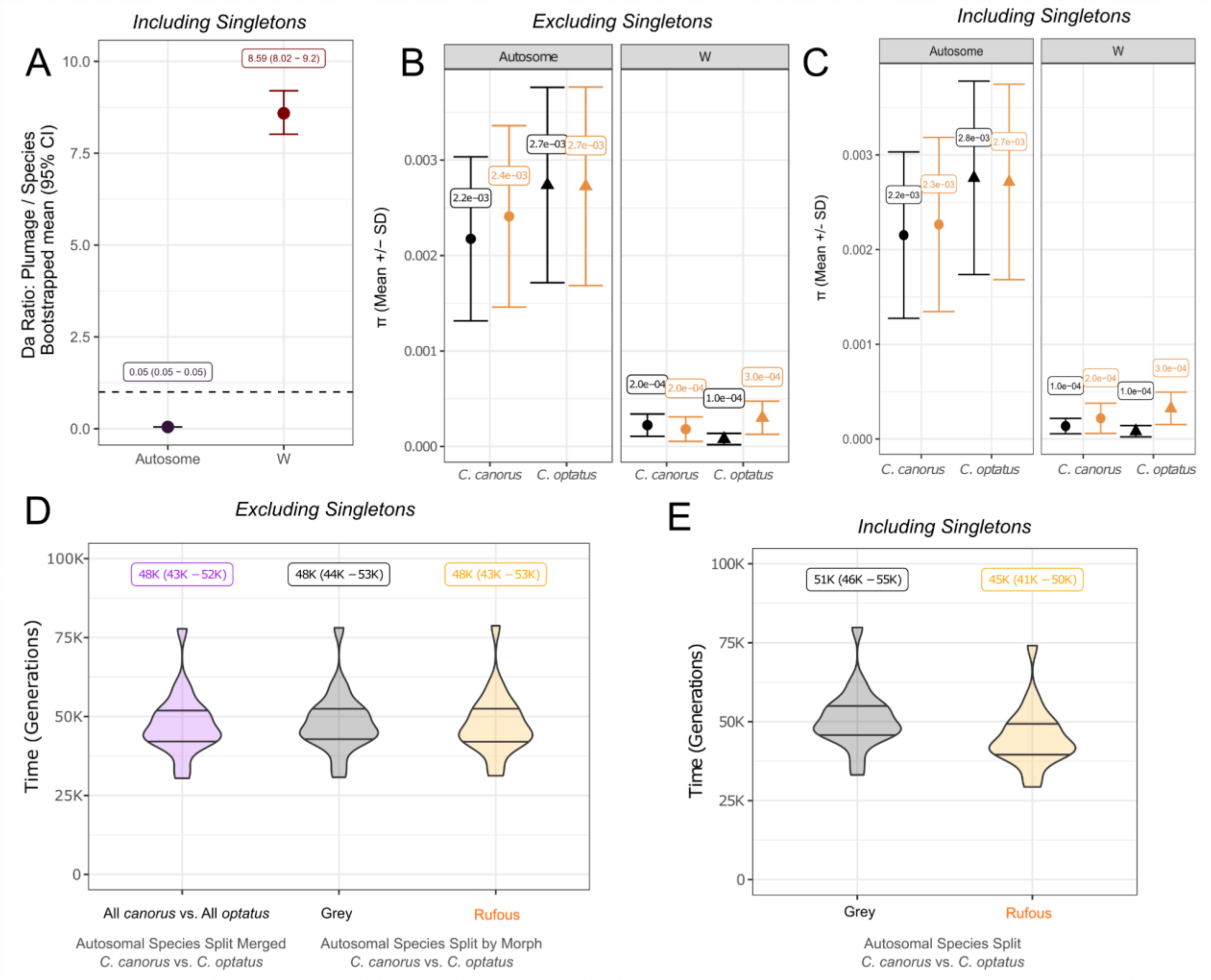

Fig. S12. Divergence dating estimates.

Insensitivity to singleton filtering (removed via bcftools –min-ac 2 in the primary figures) or morph specification for divergence estimates. The entire divergence estimation workflow was repeated both including and excluding singletons in the geno input file. Singletons could inflate genetic variation (π), particularly if many rare alleles are present in a population, so we repeated all analyses to ensure robustness. Similarly, we calculated D_a for both morphs separately (grey *canorus* vs. grey *optatus*, and rufous *canorus* vs. rufous *optatus*) and combined (all *canorus* vs. all *optatus*). (**A**) Ratio of D_a observed between plumage morphs to the D_a between species, indicating low autosomal ratios (strong species divergence) and high W chromosome ratios (strong plumage divergence). 95% confidence intervals were estimated with the boot function in *R* with 1,000 replicates. (**B**) Mean and standard deviation genetic variation (π), used directly for the D_a estimates (see methods), excluding singletons. (**C**) Same as ***(B)***, except including singletons. (**D)** Divergence time in generations considering all *canorus* vs. all *optatus and* grey and rufous morphs separately, excluding singletons. (**E**) shows divergence estimates by morph but including singletons.

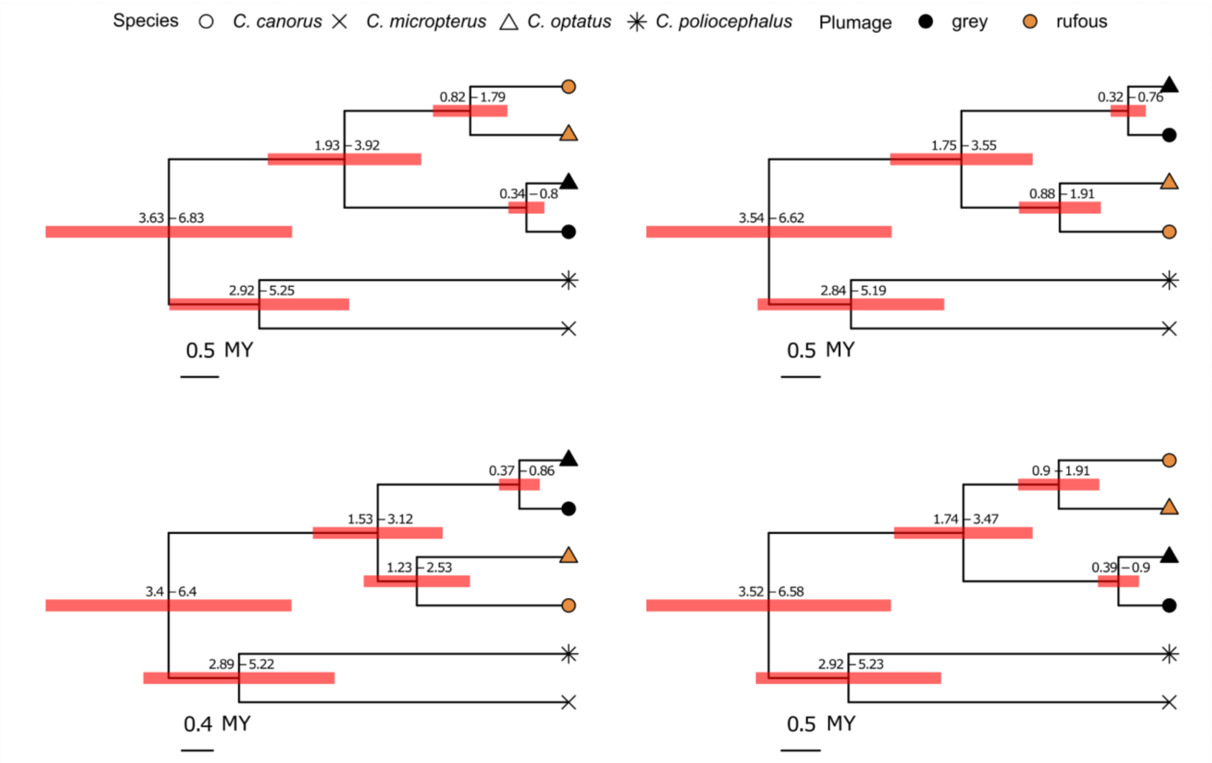

Fig. S13. Bayesian Divergence Estimation with BEAST.

Divergence dating W chromosome cuckoo morphs using BEAST v2.6.2. W chromosome consensus fasta sequences were extracted for *C. poliocephalus* and the highest coverage *C. micropterus*, as well as the five highest coverage samples from each cuckoo morph group (*C. canorus* rufous and grey, *C. optatus* rufous and grey; $n = 20$). All non-overlapping open reading frames were extracted from W chromosome genes within 10 - 75 Kb in length (same subset from the Bayesian Skyline Plots), provided that they were shared among all 22 samples and had intact start (methionine) and stop codons ($n = 513$ ORFs). BEAST input nexus files were then created by subsetting one random representative sample from each cuckoo morph group, in addition to the outgroups ($n = 6$ individuals within each BEAST analysis), to a nexus file for BEAST divergence date estimation using a Calibrated Yule model with four gamma categories and an HKY substitution model using half the autosomal mutation rate as a clock rate ($\mu_{generation} = 5.05e^{-09}$). A log normal prior for the divergence between *C. micropterus* and *C. poliocephalus* was set ($M = 1.4$, $S = 0.15$) giving an estimated 95% interval spanning 2.84 - 5.25 million years, based on estimated divergence from mtDNA data. The most supported tree was extracted and annotated with mean heights using Treeannotator with a 10% burn-in with trees and 95% HPD interval height estimates visualized with ggtree. This entire process was repeated four times to sample different representative individuals from each cuckoo morph group. Scale in millions of years (MY).

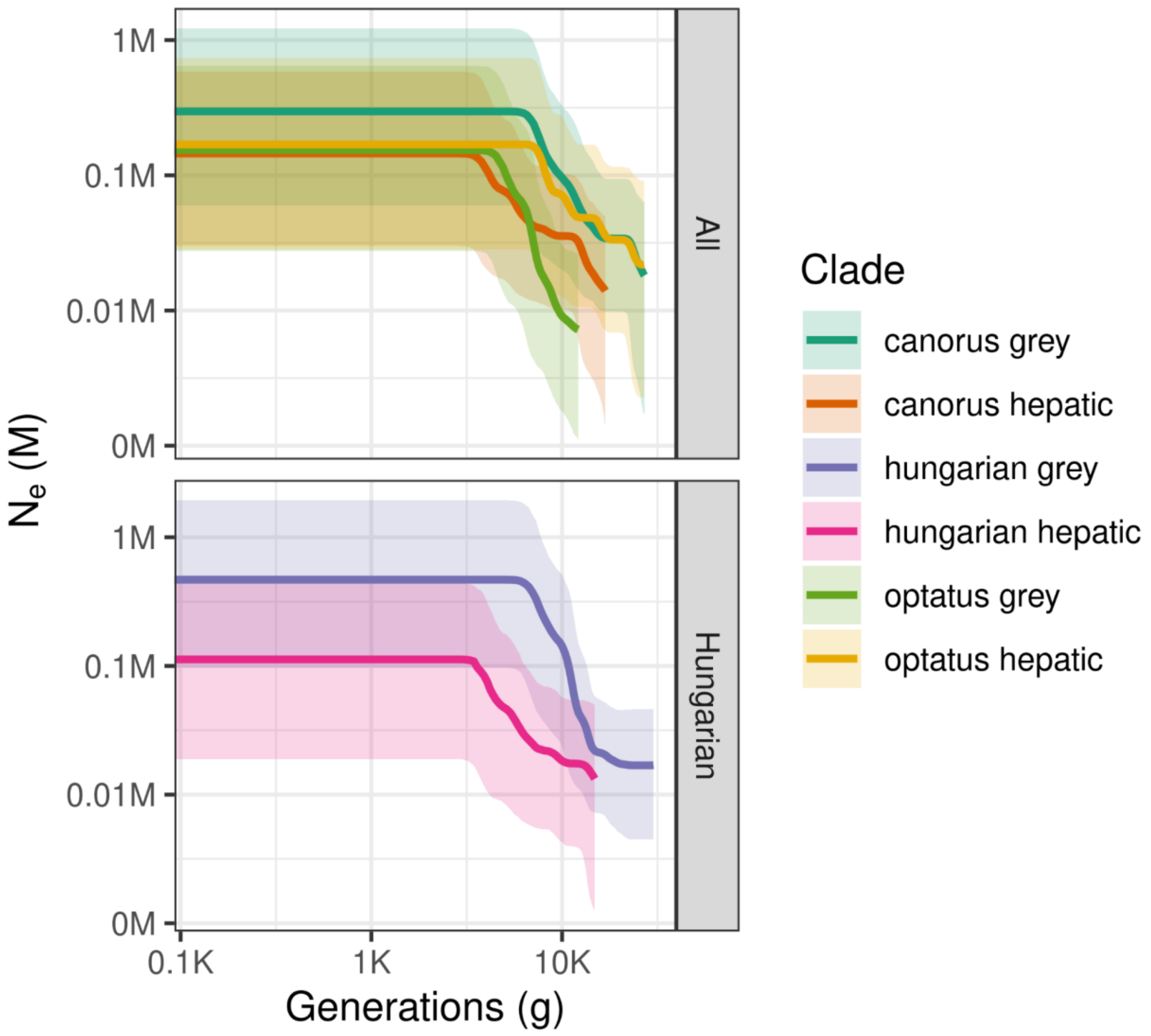

Fig. S14. Demographic history of the W chromosome.

Bayesian Skyline Plots of the female heterogametic W chromosome. Input for analyses was 1.47-Mb of W chromosome genic sequence (including introns), divided into the groups above and run with a gamma site model with four categories using a GTR substitution model with estimated frequencies. We used a strict clock set as half the autosomal generation mutation rate ($\mu_{generation}$ = 5.05e^{-09}), and ran the skyline model with 30 million chains. We ran the BSP analyses separately for the Hungarian sympatric population, in addition to the full species-morph datasets.

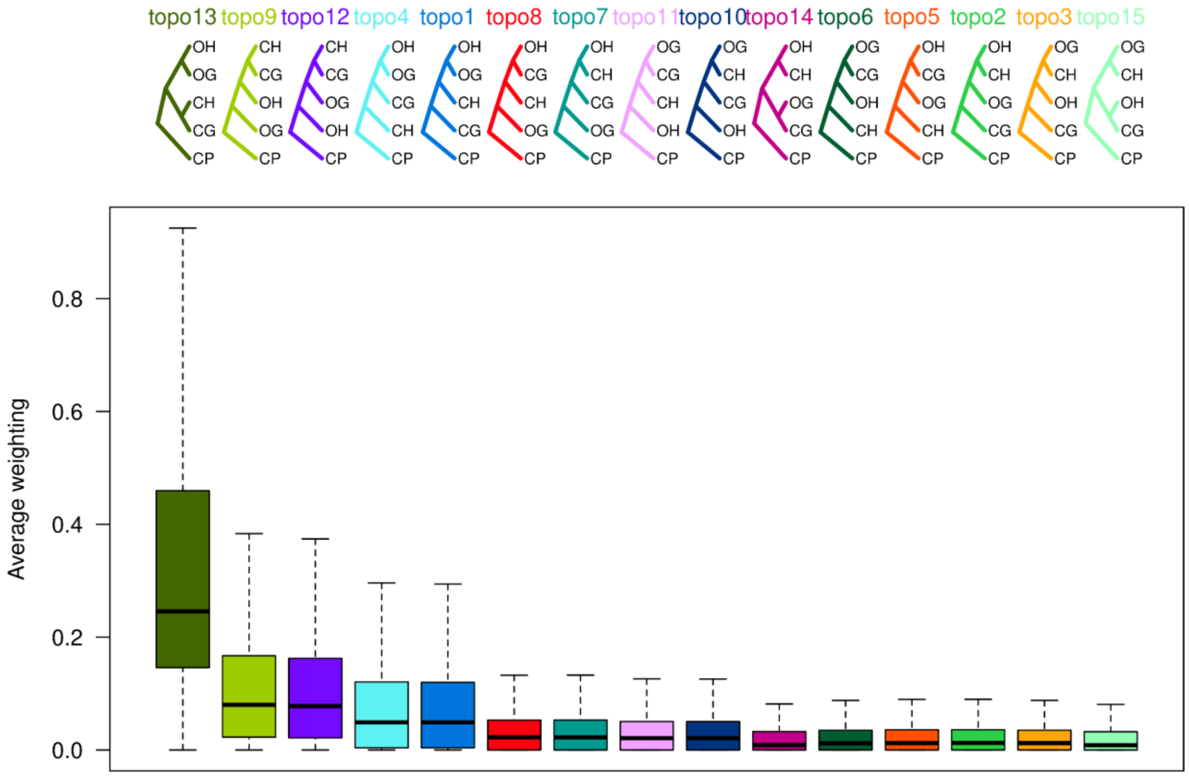

Fig. S15. Twisst whole-genome topology weights.

Topologies were estimated using phyml v3.3.20200621 wrapped in Simon Martin's genomics_general script phyml_sliding_windows.py in 100-SNP-windows genome-wide by chromosome, requiring at least 10 scored genotypes for each sample to be retained. Twisst was run as diploid for autosomes and haploid for W and Z chromosomes with *C. poliocephalus* as an outgroup and the four groups (*canorus* grey and rufous; *optatus* grey and rufous) specified as populations. Average whole-genome weights of the resulting 15 topologies are shown above as plotted using the genomics_general script plot.twisst.summary.boxplot.

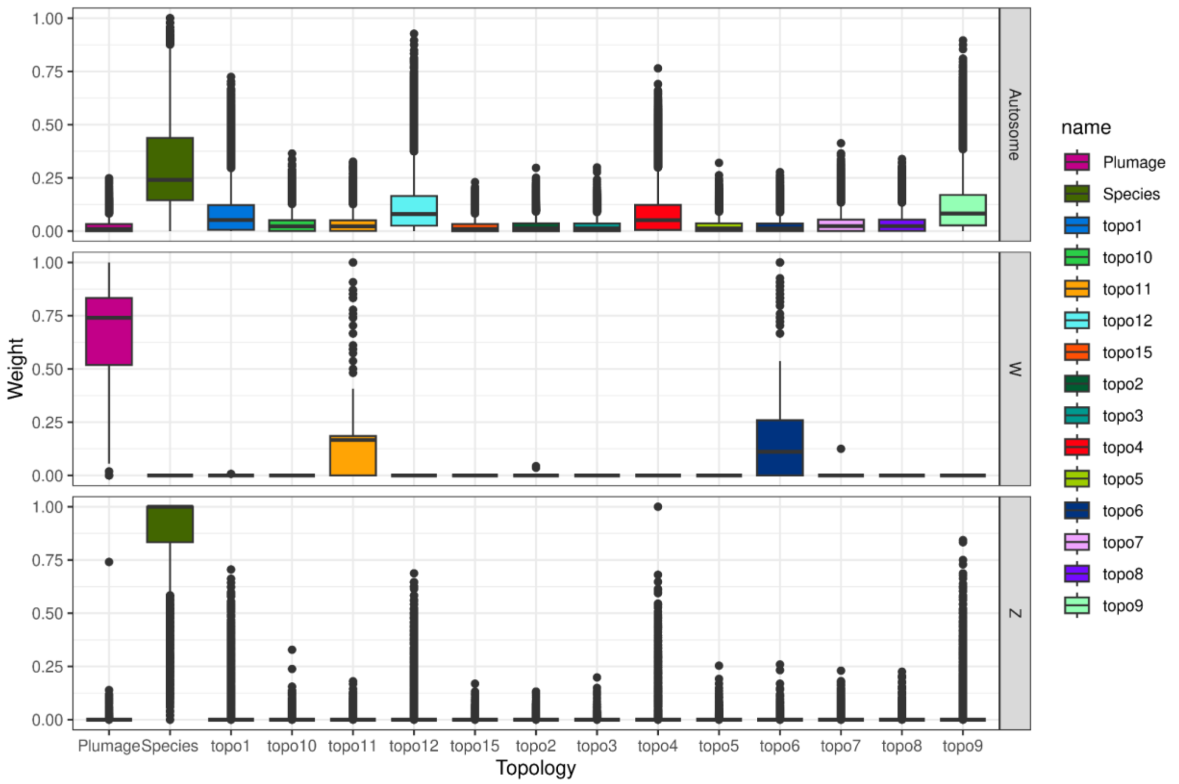

Fig. S16. Twisst topology weights by compartment.

Data is the same as shown in **Fig. S15**, but note different topology coloration from **Fig. S15**. Topologies were estimated using phyml v3.3.20200621 wrapped in Simon Martin's genomics_general script phyml_sliding_windows.py in 100-SNP-windows genome-wide by chromosome, requiring at least 10 scored genotypes for each sample to be retained. Twisst was run as diploid for autosomes and haploid for W and Z chromosomes with *C. poliocephalus* as an outgroup and the four groups (*canorus* grey and rufous; *optatus* grey and rufous) specified as populations. Average weights of the resulting 15 topologies are depicted above across each compartment using the tidyverse.

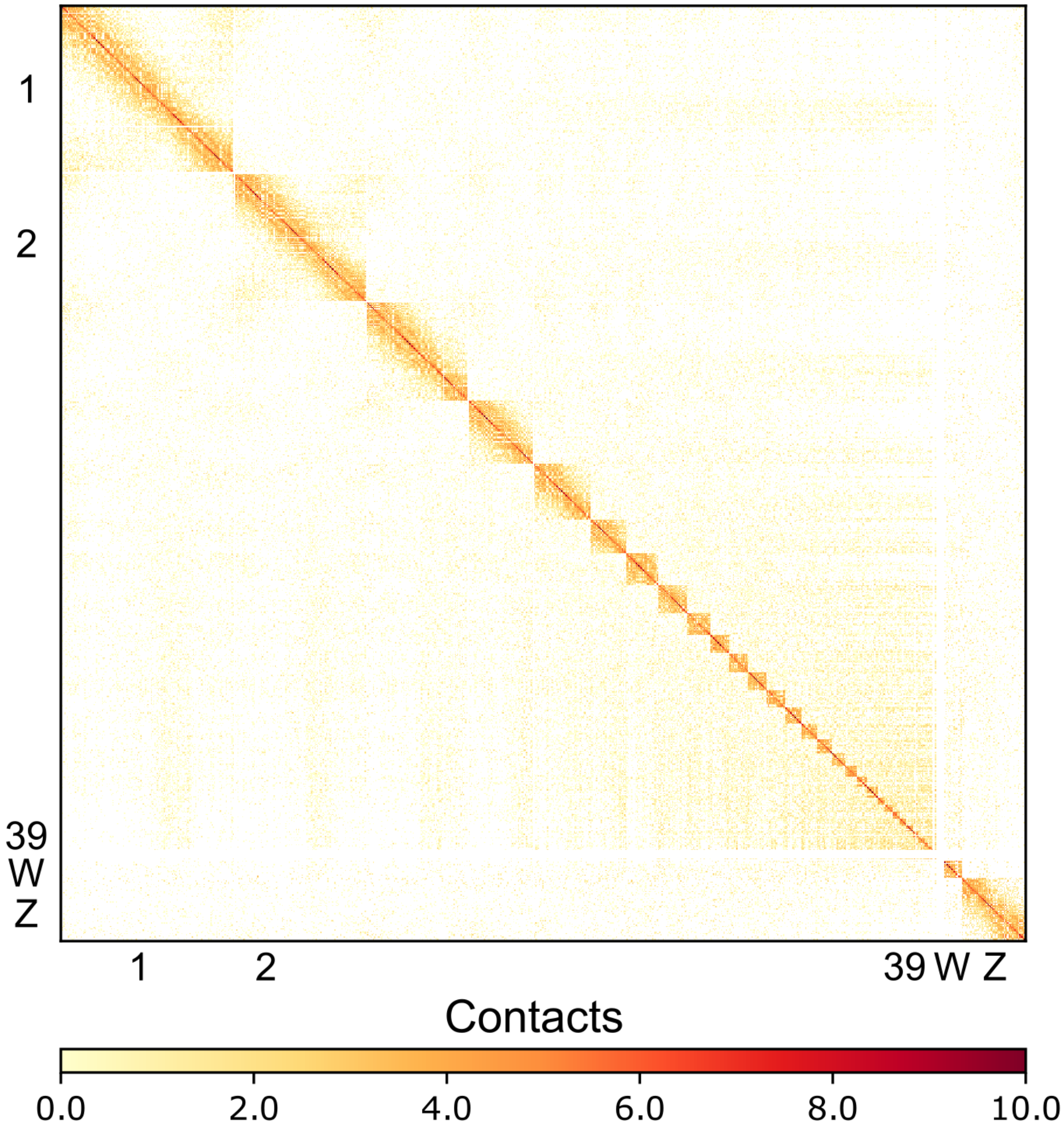

Fig. S17. Final assembly chromatin interactions.

Hi-C contact map for the final genome assembly for autosomal chromosomes 1 - 39 and the female heterogametic sex chromosomes (Z, W). Hi-C contacts were assessed using HiC-Pro from Arima Hi-C reads (Arima Genomics), here visualized in 150-kb bins.

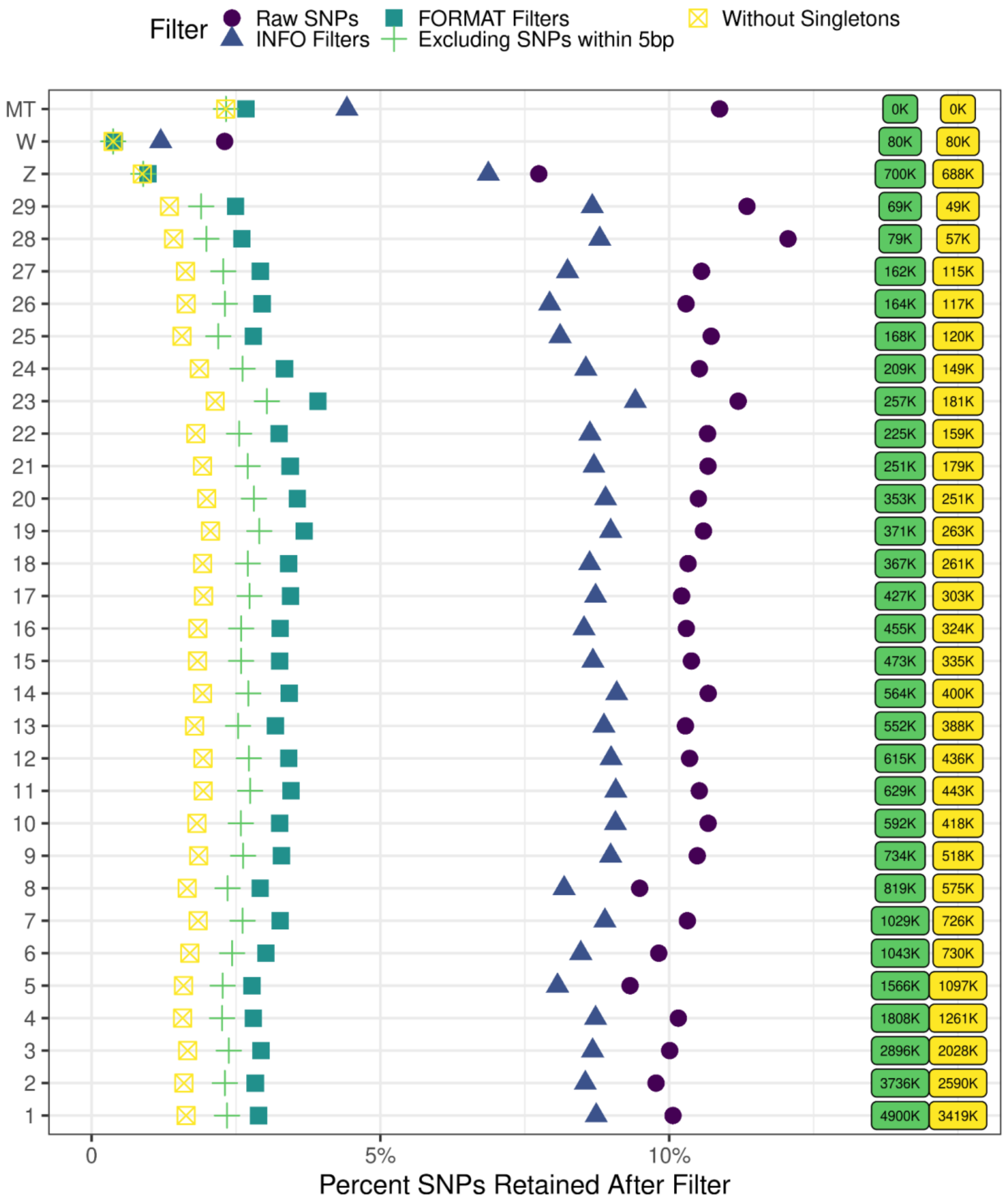

Fig. S18. SNP filtering.

Proportion of SNPs retained after each filtering step, compared to the total callable sites (variant and invariant) of each chromosome. *Raw SNPs* indicate the total SNPs from bcftools, while *INFO Filters* indicate site-level INFO filters (QUAL < 20 || INFO/DP > $DPHI || INFO/DP < $SAMPN || MQ < 30 || RPBZ < -3 || RPBZ > 3), where *$DPHI* is double the average chromosomal depth and *$SAMPN* is the number of samples. *FORMAT Filters* only retained sites with less than 20% missing genotypes, and bcftools +prune was used to retain only a single SNP from clusters with the highest MAF. The SNP set without singletons was used for PC and ADMIXTURE analyses. Labels on the left indicate final SNPs retained for the final call set (green), and final call set removing singletons (yellow).

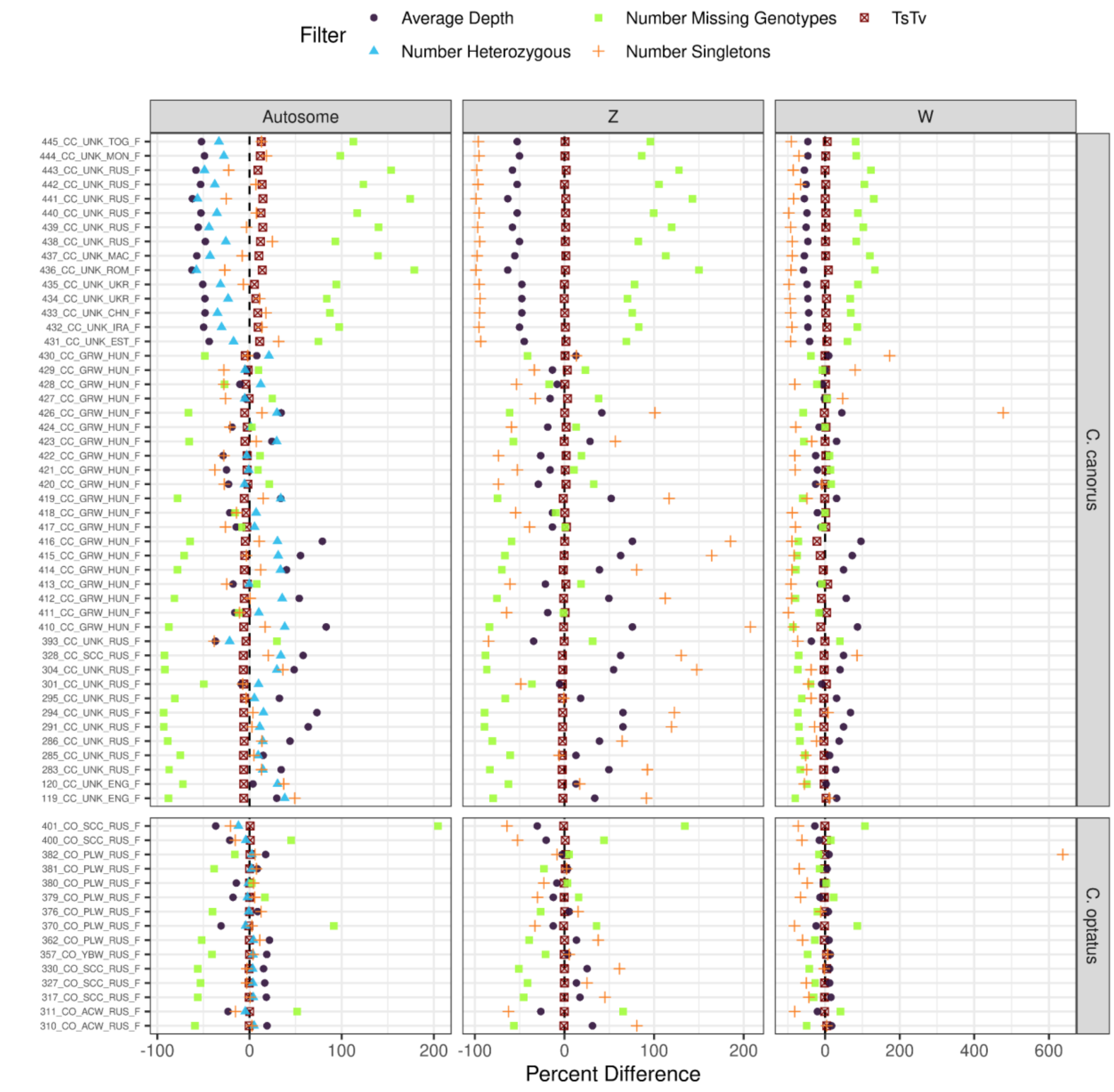

Fig. S19. SNP statistics.

SNP statistics from final call set. The mean value for each genomic compartment (autosome, W, Z) was calculated across all samples for each statistic, after which the percent difference from this average was calculated for each sample to indicate deviations. The initial 15 *C. canorus* samples are museum toepad samples which exhibit higher missing genotype rates than other samples.

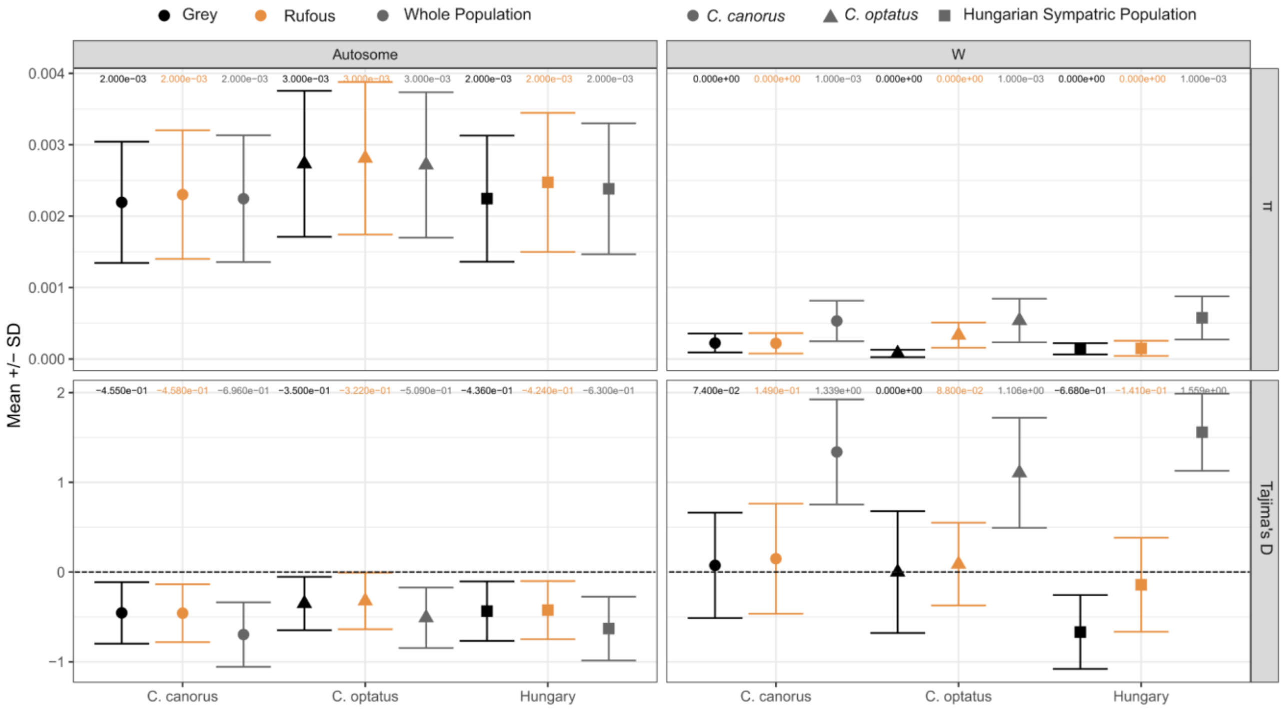

Fig. S20. Genetic variation (π) and Tajima's *D* across groups and genomic compartments. Nucleotide diversity (π) within each group in 50-kb windows was estimated with pixy (showing insensitivity to methodology), including invariant sites. Tajima's *D* was estimated using the genomics_general repository's popgenWindows.py. Mean and standard deviation were averaged across three repeated analyses choosing random samples from the indicated population at parity (*n* = 5 for morph comparisons, or *n* = 10 for All). *Hungary* indicates the sympatric Hungarian *C. canorus* population, while *C. canorus* and *C. optatus* indicate the estimates for all individuals.

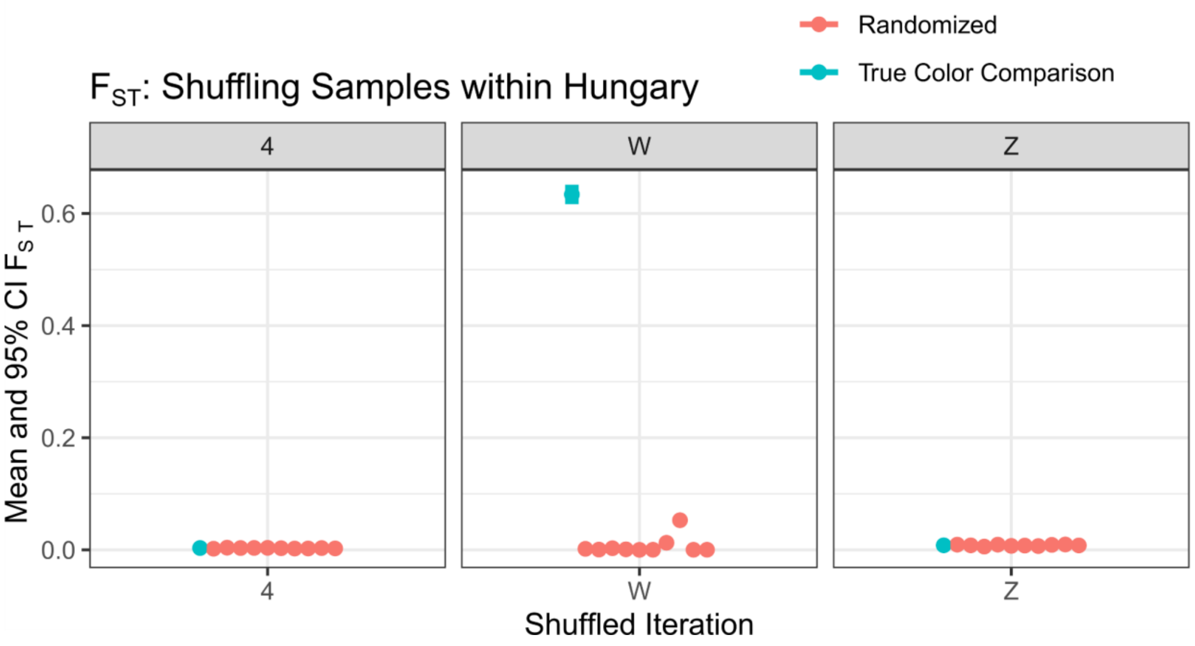

Fig. S21. Shuffled Sampling F_{ST} within Hungary.

The relationship between dichromatic differentiation and the W chromosome was confirmed with randomized shuffling of the individuals within the *Sympatric* Hungarian populations followed by F_{ST} calculation in 50-kb windows as performed in the primary manuscript. Elevated F_{ST} only for the true comparison between polychromatic morphs indicates the association between the trait and the W chromosome.

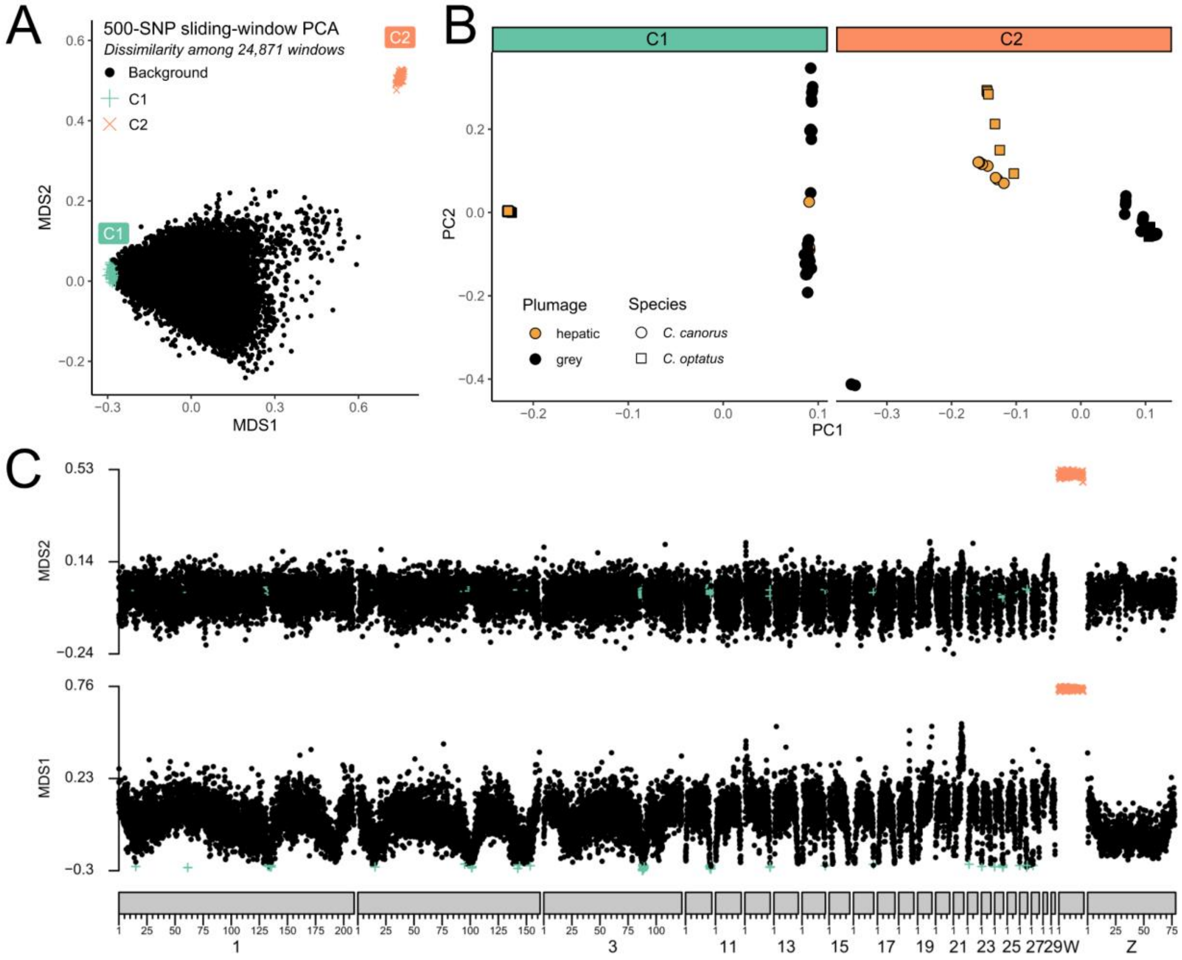

Fig. S22. Trans-specific plumage divergence without predefined groups.

Results derived from genotype data in the *Trans-species* dataset, processed through lostruct to detect dichromatic variation independent of predefined groups. The data was initially phased with imputation using Beagle v4 to ensure no missing data for PCA. Following this, genome-wide sliding window PCAs were executed in 500-SNP bins (n = 24,871 windows), with subsequent multidimensional scaling (**A**) applied to all windows to discern clusters sharing variation. In our MDS, one major outlier cluster (*C2*) and a cluster reflecting general genome-wide patterns (*C1*) were identified. Analysis of the first two axes of a PCA on windows within these specific clusters (**B**) revealed broad genome-wide patterns in *C1* driven by interspecies variation, and variation in *C2* directly correlating to plumage variation. The genome-wide visualization of MDS scores (**C**) pinpointed the W chromosome as the source of variation observed in *C2*.

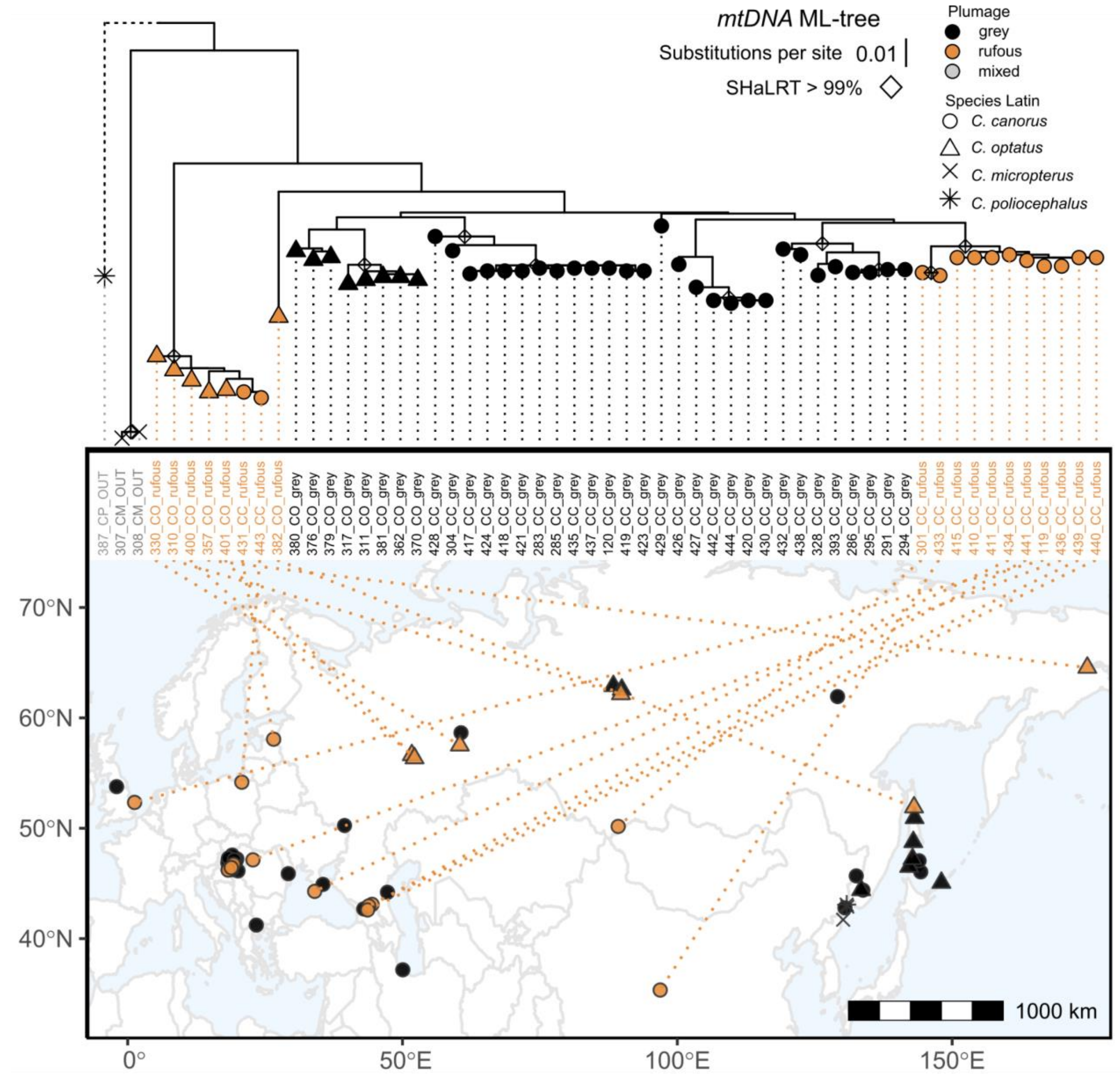

Fig. S23. Full dataset mtDNA phylogeny and spatial distribution.

Maximum-likelihood phylogeny derived from mtDNA biallelic SNPs allowing 20% missing genotypes (n = 383) using IQTREE2, modelfinderplus, and ascertainment bias correction for the full dataset, including toepad samples (n = 64; *Supporting* dataset). Low support is likely exacerbated by numerous nuclear-mitochondrial insertions throughout the autosomal genome, including a near-complete copy of the mitochondrion on chromosome 4 (**Table S8**). Given the additional complication of sequencing coverage parity between nuclear DNA and mtDNA in nucleated blood libraries, the W chromosome was preferred for evolutionary inference.

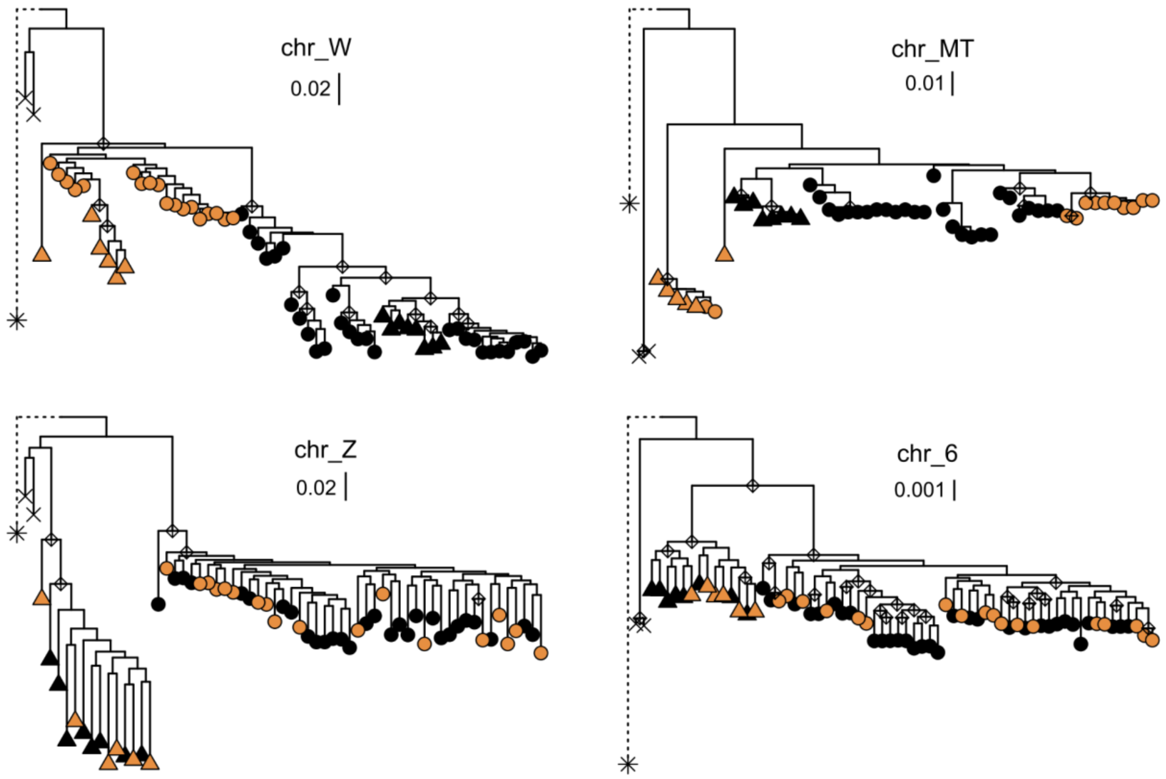

Fig. S24. Phylogenetic Trees for all chromosomes.

Maximum-likelihood phylogenies for W chromosome (*n* = 5,155), mtDNA (*n* = 375), Z chromosome (*n* = 10,811), and chromosome 6 (*n* = 799,615) for the *Supporting* dataset, with strict missing data filters (maximum 5% missing genotypes). Trees were made using IQTREE using modelfinderplus with ascertainment bias correction. Nodes with SH-approximation likelihood ratio test (*SH-aLRT*) support values > 99% are indicated with a diamond. As all individuals are female, the W, Z, and mtDNA were ensured to be genotyped as haploid prior to tree generation (see methods).

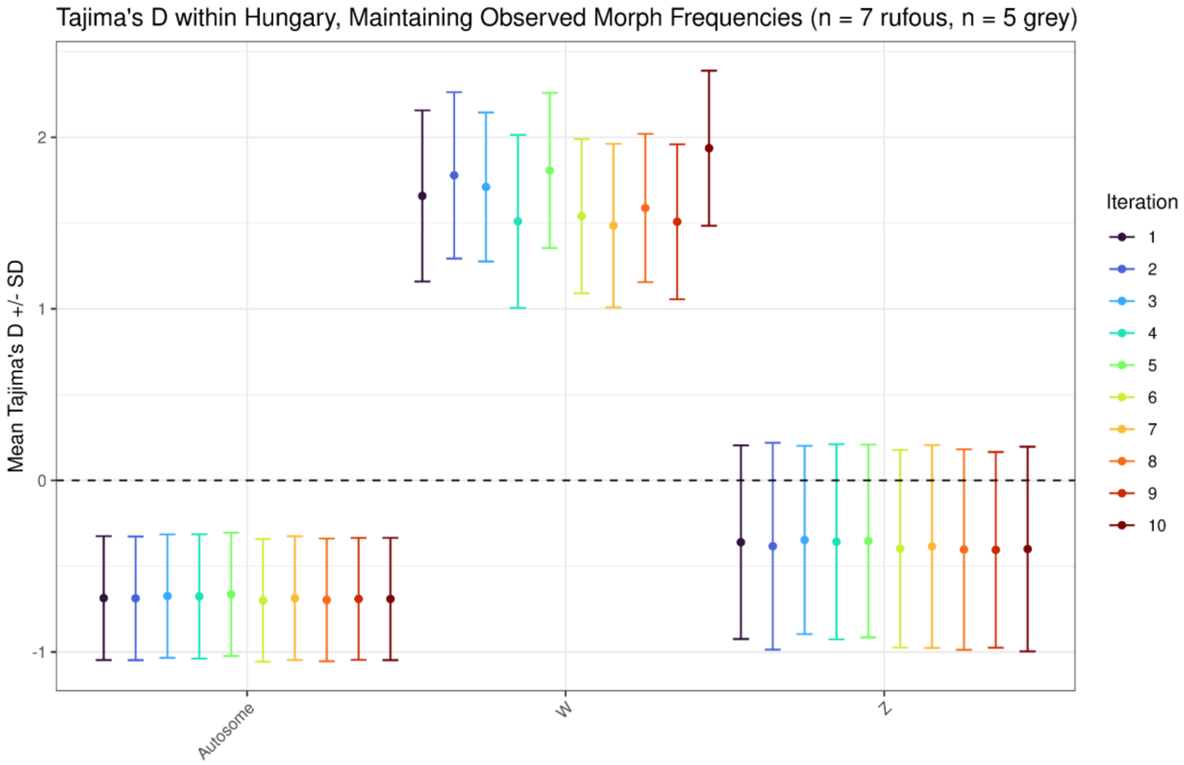

Fig. S25. Tajima's *D* with Observed Morph Frequencies.

Tajima's *D* was estimated using the genomics_general repository's popgenWindows.py, including singletons. In addition to reporting Tajima's D for the *Sympatric* Hungarian *C. canorus* population with sampling at parity (*n = 5* for rufous and grey morphs), here Tajima's *D* is estimated at the observed population morph frequencies (60% rufous, 40% grey), by randomly subsampling respective individual morphs (without replacement) and calculating Tajima's *D* in 50-kb windows for the subset population, including both morphs, repeated 10 times (iterations).

Supplementary Tables

Table S2. Genome assembly statistics.

RefSeq Genome assembly statistics for the rufous *C. canorus* generated in this study (GCF_017976375.1), obtained from the NCBI dashboard.

Variable	Value
Genome size	1.2-Gb
Total un-gapped length	1.2-Gb
Number of chromosomes	41
Number of scaffolds	150
Scaffold N50	78.3-Mb
Scaffold L50	5
Number of contigs	446
Contig N50	20.8-Mb
Contig L50	17
GC percent	42
Genome coverage	64.3x
Assembly level	Chromosome
Genes	19729
Protein-coding Genes	16774
Non-coding Genes	2741
BUSCO Single-copy	98.4
BUSCO Duplicated	0.5
BUSCO Fragmented	0.2
BUSCO Missing	0.9
Chromosome Z Length	78.3-Mb
Chromosome W Length	22.3-Mb

Table S3. Species and plumage associations with global autosomal and W chromosomal variation.

Significance for species and plumage effects for the first 6 axes of a PCA summarizing SNP variation for autosomal (*n* = 16,031,301 SNPs) and W chromosome (*n* = 53,712) biallelic SNP data. For each dataset (autosomes, W chromosome) and each PC axis, two Wilcoxon rank-sum tests were performed followed by Bonferonni correction (*n* = 24). Owing to normality violations we were restricted to non-parametric statistics.

PC Axis	Analysis	Species (Wilcoxon *p*-adjusted)	Plumage (Wilcoxon *p*-adjusted)
1	Autosomes	$2.13E^{-12}$	1.00
2	Autosomes	0.0510	1.00
3	Autosomes	1.00	0.243
4	Autosomes	1.00	1.00
5	Autosomes	0.354	1.00
6	Autosomes	1.00	1.00
1	W Chromosome	1.00	$8.08E^{-16}$
2	W Chromosome	1.00	1.00
3	W Chromosome	0.00171	0.232
4	W Chromosome	1.00	0.832
5	W Chromosome	1.00	0.0222
6	W Chromosome	1.00	1.00

Table S4. Divergence dating between species and plumage comparisons.

Time in generations was estimated using mutations accumulated since divergence from a common ancestor (D_a), estimated using pairwise divergence (D_{XY}, calculated using invariant sites), and variation observed within each group (π), see methods for further details. Mean D_a and 95% bootstrapped confidence intervals are shown, with time to generations calculated using the per-generation neutral substitution rate ($\mu_{generation}$ = 1.01e^{-08}). Generation times were rounded to the nearest whole number for readability. Hungarian sympatric population plumage comparison is indicated as a distinct comparison. This table shows D_a estimates for D_{XY} and π calculated for all samples.

**Compartment**	**Group**	D_a	D_a **Lower (95% CI)**	D_a **Upper (95% CI)**	**Time Mean (Generations)**	**Time Lower (Generations, 95% CI)**	**Time Upper (Generations, 95% CI)**	**Facet**
Autosome	All *canorus* vs. All *optatus*	9.63E^{-04}	8.69E^{-04}	1.06E^{-03}	47679	42996	52363	Species Comparison
Autosome	*canorus* grey vs. *optatus* grey	9.62E^{-04}	8.25E^{-04}	1.10E^{-03}	47904	41502	54307	Species Comparison
Autosome	*canorus* rufous vs. *optatus* rufous	9.62E^{-04}	8.25E^{-04}	1.10E^{-03}	47735	41070	54399	Species Comparison
Autosome	*canorus* grey vs. *canorus* rufous	3.93E^{-05}	0.00	1.01E^{-04}	3432	0.00	9572	Plumage Comparison
Autosome	Hungary *canorus* grey vs. Hungary *canorus* rufous	3.31E^{-05}	0.00	9.82E^{-05}	3192	0.00	9637	Plumage Comparison
Autosome	*optatus* grey vs. *optatus* rufous	5.23E^{-05}	0.00	1.09E^{-04}	3999	0.00	9755	Plumage Comparison

Table S5. Topology weighting to assess the effects of incomplete lineage sorting.

Each chromosome was divided into 100 SNP-windows (requiring at least 10% genotypes per individual) followed by maximum-likelihood tree estimation with phyml using a GTR substitution model on each window. Twisst was then used with *C. poliocephalus* as an outgroup to assess topology weighting of the 15 possible topologies given the four populations: *C. canorus* grey and rufous, and *C. optatus* grey and rufous. Topology weights were divided by compartment (autosome, Z, W) and trees were assigned to either the 'Species' tree, the 'Plumage' tree, or an incomplete lineage sorting tree 'ILS', which is the cumulative weights of topologies not including the ‘Species’ or ‘Plumage’ trees. Summary statistics of these weights for these trees and compartments are below. Windows were assigned a 'majority support' if the topology weight of ‘Species’, ‘Plumage’, or ‘ILS’ was greater than or equal to 50%. Window size in bases differed among the compartments, and is indicated. 1,000 bootstrap replicates were performed to estimate majority topology lower and upper confidence intervals.

**Topology**	**Compartment**	**Total Windows**	**Weight (Mean)**	**Weight (StdDev)**	**Number Windows With Majority (>= 0.5)**	**Proportion Windows Showing Majority Topology**	**Proportion (Lower 95%)**	**Proportion (Upper 95%)**	**Average Window Size (BP)**	**Analysis Window Size (SNPs)**
ILS	Autosome	173,231	0.640	0.253	136,440	0.788	0.786	0.790	6,030	100
Plumage	Autosome	173,231	0.022	0.030	0.00	0.00	0.00	0.00	6,030	100
Species	Autosome	173,231	0.338	0.265	36,355	0.210	0.208	0.212	6,030	100
ILS	W Chromosome	539	0.364	0.326	121	0.224	0.189	0.260	39,275	100
Plumage	W Chromosome	539	0.636	0.326	419	0.777	0.742	0.813	39,275	100
Species	W Chromosome	539	0.00	0.00	0.00	0.00	0.00	0.00	39,275	100
ILS	Z Chromosome	4,512	0.146	0.237	568	0.126	0.115	0.135	17,172	100
Plumage	Z Chromosome	4,512	0.001	0.013	1.00	0.00	0.00	0.001	17,172	100
Species	Z Chromosome	4,512	0.853	0.240	3,967	0.879	0.87	0.889	17,172	100

Table S6. Jack-knifed *D*-statistics from genome-wide autosomal ABBA-BABA analyses. Excess allele sharing between *C. optatus* rufous and both *C. canorus* rufous and grey was assessed by repeating the jack-knifed *D*-statistical analysis twice with *C. canorus* grey as *P3* and *C. canorus* rufous as *P3*, with *C. poliocephalus* as the outgroup (*P4*), *C. optatus* grey as *P1*, and *C. optatus* rufous as *P2*. Frequencies and estimates were inferred using the genomics_general repository.

P1	***P2***	***P3***	***O***	***D* Jackknife (Mean)**	***D* Jackknife (Variance)**	***D* Jackknife (SD)**	***D* Jackknife (SE)**	***Z*-score**	**Admixture Proportion (*f*)**	**Dataset**
C. optatus grey	*C. optatus rufous*	*C. canorus grey*	*C. poliocephalus*	0.0177	0.0055	0.074	0.002	7.84	0.0184	*Hungarian Sympatric C. canorus*
C. optatus grey	*C. optatus rufous*	*C. canorus rufous*	*C. poliocephalus*	0.0181	0.0055	0.074	0.002	8.03		*Hungarian Sympatric C. canorus*
C. optatus grey	*C. optatus rufous*	*C. canorus grey*	*C. poliocephalus*	0.0166	0.0044	0.066	0.002	8.17	0.0171	*Supporting Dataset*
C. optatus grey	*C. optatus rufous*	*C. canorus rufous*	*C. poliocephalus*	0.0166	0.0044	0.066	0.002	8.20		*Supporting Dataset*
C. optatus grey	*C. optatus rufous*	*C. canorus grey*	*C. poliocephalus*	0.0166	0.0044	0.067	0.002	8.14	0.0170	*Trans-species Dataset*
C. optatus grey	*C. optatus rufous*	*C. canorus rufous*	*C. poliocephalus*	0.0163	0.0044	0.067	0.002	8.03		*Trans-species Dataset*

Table S7. Genetic variation and Tajima's *D* genome-wide.

Tajima's D and π were calculated in 50-Kb windows genome-wide using the genomics_general repository from an all-sites geno file including invariant sites, using the *Trans-species* dataset. Sensitivity to sampling was assessed by randomly subsampling 5 individuals from each morph and repeating the analysis for a total of 3 times. All comparisons included 5 randomly subsampled morphs of either respective population. Values indicate the summary statistics across all 3 iterations. Rows labelled *Hungary* indicate variation only within the sympatric Hungarian *C. canorus* population.

Population	Morph	Compartment	Variable	Mean	Standard Deviation	Median	IQR	Lower 95% CI	Upper 95% CI
C. canorus	Grey	Autosome	π	0.0022	0.0009	0.0022	0.0012	0.0022	0.0022
C. canorus	Rufous	Autosome	π	0.0024	0.0010	0.0024	0.0013	0.0024	0.0024
C. canorus	All	Autosome	π	0.0023	0.0009	0.0024	0.0013	0.0023	0.0024
C. optatus	Grey	Autosome	π	0.0027	0.0010	0.0028	0.0014	0.0027	0.0027
C. optatus	Rufous	Autosome	π	0.0027	0.0010	0.0028	0.0014	0.0027	0.0027
C. optatus	All	Autosome	π	0.0028	0.0010	0.0028	0.0014	0.0027	0.0028
Hungary	Grey	Autosome	π	0.0023	0.0009	0.0023	0.0012	0.0022	0.0023
Hungary	Rufous	Autosome	π	0.0025	0.0010	0.0025	0.0013	0.0024	0.0025
Hungary	All	Autosome	π	0.0024	0.0009	0.0024	0.0012	0.0024	0.0024
C. canorus	Grey	W	π	0.0002	0.0001	0.0002	0.0002	0.0002	0.0002
C. canorus	Rufous	W	π	0.0002	0.0001	0.0002	0.0001	0.0002	0.0002
C. canorus	All	W	π	0.0006	0.0003	0.0006	0.0003	0.0006	0.0006
C. optatus	Grey	W	π	0.0001	0.0001	0.0001	0.0001	0.0001	0.0001
C. optatus	Rufous	W	π	0.0003	0.0002	0.0003	0.0002	0.0003	0.0003
C. optatus	All	W	π	0.0006	0.0003	0.0006	0.0003	0.0005	0.0006
Hungary	Grey	W	π	0.0001	0.0001	0.0002	0.0001	0.0001	0.0002
Hungary	Rufous	W	π	0.0002	0.0001	0.0002	0.0001	0.0002	0.0002
Hungary	All	W	π	0.0006	0.0003	0.0006	0.0003	0.0006	0.0006
C. canorus	Grey	Z	π	0.0009	0.0006	0.0008	0.0008	0.0009	0.0009
C. canorus	Rufous	Z	π	0.0010	0.0007	0.0009	0.0009	0.0010	0.0011
C. canorus	All	Z	π	0.0010	0.0006	0.0009	0.0009	0.0010	0.0010
C. optatus	Grey	Z	π	0.0014	0.0004	0.0013	0.0005	0.0014	0.0014
C. optatus	Rufous	Z	π	0.0014	0.0005	0.0014	0.0006	0.0014	0.0014
C. optatus	All	Z	π	0.0014	0.0005	0.0014	0.0006	0.0014	0.0014
Hungary	Grey	Z	π	0.0009	0.0006	0.0008	0.0008	0.0009	0.0009
Hungary	Rufous	Z	π	0.0011	0.0007	0.0010	0.0009	0.0011	0.0011
Hungary	All	Z	π	0.0010	0.0006	0.0009	0.0009	0.0010	0.0010
C. canorus	Grey	Autosome	Tajima's D	-0.4549	0.3428	-0.4219	0.3333	-0.4576	-0.4522
C. canorus	Rufous	Autosome	Tajima's D	-0.4583	0.3228	-0.4289	0.3097	-0.4608	-0.4558
C. canorus	All	Autosome	Tajima's D	-0.6964	0.3595	-0.6486	0.3546	-0.6992	-0.6936
C. optatus	Grey	Autosome	Tajima's D	-0.3503	0.2973	-0.3452	0.2779	-0.3526	-0.3479
C. optatus	Rufous	Autosome	Tajima's D	-0.3218	0.3152	-0.3183	0.2920	-0.3242	-0.3193
C. optatus	All	Autosome	Tajima's D	-0.5092	0.3370	-0.4908	0.3065	-0.5118	-0.5066

Hungary	Grey	Autosome	Tajima's D	-0.4358	0.3311	-0.4110	0.3273	-0.4384	-0.4332
Hungary	Rufous	Autosome	Tajima's D	-0.4241	0.3245	-0.4023	0.3162	-0.4266	-0.4216
Hungary	All	Autosome	Tajima's D	-0.6302	0.3556	-0.5929	0.3499	-0.6329	-0.6274
C. canorus	Grey	W	Tajima's D	0.0744	0.5873	0.0830	0.8138	0.0412	0.1076
C. canorus	Rufous	W	Tajima's D	0.1490	0.6130	0.1910	0.9110	0.1140	0.1835
C. canorus	All	W	Tajima's D	1.3387	0.5857	1.3314	0.8131	1.3057	1.3718
C. optatus	Grey	W	Tajima's D	-0.0003	0.6779	0.0000	0.9677	-0.0386	0.0380
C. optatus	Rufous	W	Tajima's D	0.0884	0.4613	0.0849	0.5633	0.0624	0.1145
C. optatus	All	W	Tajima's D	1.1057	0.6132	1.1323	0.7624	1.0711	1.1404
Hungary	Grey	W	Tajima's D	-0.6680	0.4113	-0.7468	0.5372	-0.6912	-0.6447
Hungary	Rufous	W	Tajima's D	-0.1412	0.5240	-0.1909	0.7126	-0.1708	-0.1116
Hungary	All	W	Tajima's D	1.5586	0.4299	1.5978	0.5035	1.5343	1.5829
C. canorus	Grey	Z	Tajima's D	-0.1167	0.4856	-0.0939	0.5757	-0.1307	-0.1027
C. canorus	Rufous	Z	Tajima's D	-0.1682	0.4430	-0.1420	0.5261	-0.1809	-0.1555
C. canorus	All	Z	Tajima's D	-0.3054	0.6153	-0.2546	0.7041	-0.3231	-0.2877
C. optatus	Grey	Z	Tajima's D	-0.0215	0.2105	-0.0274	0.2514	-0.0276	-0.0154
C. optatus	Rufous	Z	Tajima's D	-0.0662	0.2188	-0.0652	0.2690	-0.0725	-0.0599
C. optatus	All	Z	Tajima's D	-0.0640	0.3050	-0.0661	0.3654	-0.0728	-0.0552
Hungary	Grey	Z	Tajima's D	-0.1045	0.4603	-0.0780	0.5490	-0.1177	-0.0913
Hungary	Rufous	Z	Tajima's D	-0.1921	0.4237	-0.1575	0.5194	-0.2043	-0.1800
Hungary	All	Z	Tajima's D	-0.3028	0.5541	-0.2443	0.6747	-0.3188	-0.2869

Table S8. Nuclear mitochondrial insertions (NuMTs) in the cuckoo chromosome-level assembly.

We identified NuMTs by blasting the whole nucleotide sequence of the cuckoo mitochondrion against the entire assembly using blastn v2.2.31+. We then merged any hits within 500-bp and averaged the percent identity from those respective hits into the coordinates listed below. The self-hit against the mitochondrion is listed for reference.

Chromosome	Start	End	Percent Identity	Strand	Length
mtDNA	1	19698	100	Positive	19698
4	61666901	61684920	90.5075	Negative	18020
W	20151478	20157090	85.9	Positive	5613
W	20126237	20129515	86.67	Negative	3279
2	73876144	73879209	84.69	Negative	3066
W	9904691	9906685	88.64	Positive & Negative (multiple overlaps)	1995
W	16827543	16829041	93.03	Negative	1499
W	1.68E+07	1.68E+07	90.27	Negative	1206
1	1.91E+08	1.91E+08	75.45	Positive	926
3	6127172	6127934	88.99	Negative	763
1	81069613	81070111	81.98	Positive	499
Z	70598461	70598807	96.57	Negative	347
2	51908944	51909249	88.96	Positive	306
Z	63366061	63366304	84.49	Negative	244
12	12056647	12056674	100	Positive	28

Table S9. Estimated contemporary and overall averaged effective population size (*N_e*) of the W chromosome from Bayesian Skyline Plot analyses.

Input for analyses was 1.47-Mb of W chromosome genic sequence (including introns), divided into the groups indicated below, and run with a gamma site model with 4 categories and a GTR substitution model with estimated frequencies. We used a strict clock set as half the autosomal generation mutation rate ($\mu_{generation}$ = 5.05e^{-09}), and ran the skyline model with 30 million chains. Contemporary estimates of mean, median, and 95% confidence intervals come from the Bayesian Skyline Plot results at time point 0, while the 'Averaged All' estimates show a summary of the mean, median, and 95% CI of the mean N_e observed across all time points.

Time	Mean	Median	Upper	Lower	*ESS* (posterior)	Clade	Morph	Sample Selection
0 (Contemporary)	296900	184710	32978.7	844530	9001	*canorus*	grey	All Samples
0 (Contemporary)	145030	88404.4	14910.4	402890	8258.9	*canorus*	hepatic	All Samples
0 (Contemporary)	153380	87799.1	15699.9	435650	8762.1	*optatus*	grey	All Samples
0 (Contemporary)	169040	95519.6	13079.4	486260	8946	*optatus*	hepatic	All Samples
0 (Contemporary)	468220	292270	59338.9	1308900	8817.4	*canorus*	grey	Hungarian
0 (Contemporary)	112440	60114.7	11375.9	297470	8652	*canorus*	hepatic	Hungarian
Averaged All	117637.6	46328.1	5344.8	139930.4	-	*canorus*	grey	All Samples
Averaged All	62825.4	38194.2	53212.9	72437.9	-	*canorus*	hepatic	All Samples
Averaged All	78248.6	65143.1	65552.7	90944.4	-	*optatus*	grey	All Samples
Averaged All	79252.6	48503.2	67497.9	91007.3	-	*optatus*	hepatic	All Samples
Averaged All	148330.7	21622.5	111397	185264.4	-	*canorus*	grey	Hungarian
Averaged All	47926.1	22903.2	40065.2	55787	-	*canorus*	hepatic	Hungarian

Table S10. Divergence dating W chromosomes using *BEAST*.

W chromosome consensus fasta sequences were extracted for female *C. poliocephalus* and the highest coverage *C. micropterus* samples, as well as the 5 highest coverage samples from each cuckoo morph group (*C. canorus* rufous and grey, *C. optatus* rufous and grey; *n* = 20). All non-overlapping open reading frames were extracted from W chromosome genes within 10 – 75 Kb in length (same subset from the Bayesian Skyline Plots, above), provided that they were shared among all 22 samples and had intact start (methionine) and stop codons (*N* = 513 ORFs). At this point, we subset 1 random representative sample from each cuckoo morph group, in addition to the outgroups (*N* = 6 individuals within each BEAST analysis), to a nexus file for BEAST divergence date estimation using a Calibrated Yule model with 4 gamma categories and an HKY substitution model using half the autosomal mutation rate as a clock rate ($\boldsymbol{\mu}_{generation}$ = 5.05e^{-09}). A log normal prior for the divergence between *C. micropterus* and *C. poliocephalus* was set (*M* = 1.4, *S* = 0.15) giving an estimated 95% interval spanning 2.84 - 5.25 million years, based on estimated divergence from mtDNA data. The best supported tree was extracted and annotated with mean heights using Treeannotator with a 10% burn-in with trees and 95% HPD interval height estimates visualized with ggtree. This entire process was repeated 4 times to sample different representative individuals from each cuckoo morph group.

**Iteration**	**ESS (posterior)**	**Tree Height (Mean)**	**Tree Height (SD)**	**Divergence *C. micropterus* - *C. poliocephalus* (MYA; 95% HPD interval)**	**Divergence Grey & Rufous Morphs (MYA; 95% HPD interval)**
1	27,491.70	7.74	1.61	2.92 - 5.25	1.93 - 3.92
2	26,370.00	7.52	1.56	2.84 - 5.19	1.53 - 3.12
3	26,538.90	7.20	1.49	2.89 - 5.22	1.75 - 3.55
4	27,469.10	7.31	1.51	2.92 - 5.23	1.74 - 3.47

External Table S1

Table S1 (Sample Metadata; TableS1.xlsx)
